# Supplementary material for: An energetics-based honeybee nectar-foraging model used to assess the potential for landscape-level pesticide exposure dilution
Source: PeerJ. 2016 Aug 16;4:e2293. doi: 10.7717/peerj.2293 (PMC4991850; doi:10.7717/peerj.2293)
Supplement: Supplemental Information 1 [file peerj-04-2293-s001.pdf]

Supplementary Information - Supporting Data

Title: An energetics-based honeybee nectar-foraging model used to assess the potential for landscape-level pesticide exposure dilution

Johannes M. Baveco, Alterra, Wageningen University & Research Center, Wageningen, The Netherlands

Andreas Focks, Alterra, Wageningen University & Research Center, Wageningen, The Netherlands

Dick Belgers, Alterra, Wageningen University & Research Center, Wageningen, The Netherlands

Jozef J.M. van der Steen, Plant Research International, Wageningen University & Research Center, Wageningen, The Netherlands

Jos J.T.I. Boesten, Alterra, Wageningen University & Research Center, Wageningen, The Netherlands

Ivo Roessink, Alterra, Wageningen University & Research Center, Wageningen, The Netherlands

Estimates of attack-rate for clover and oilseed rape

Clover

Data from (Goodwin et al. 2011)

MathCad code:

$\mathrm{flDensity} := 2808$ $\qquad \mathrm{flPerSec} := \frac{3.79}{11.33}$ $\qquad h := 1.215$ $\qquad$ handling time per floret (s)

$$\mathrm{GF(alpha)} := (\mathrm{flPerSec}) - \left(\mathrm{alpha} \cdot \frac{\mathrm{flDensity}}{1 + \mathrm{alpha} \cdot \mathrm{flDensity} \cdot h}\right)$$

$\mathrm{alpha} := 0.0001$

$\mathrm{acalc} := \mathrm{root}(\mathrm{GF(alpha)}, \mathrm{alpha})$

$\mathrm{acalc} = 2.007 \times 10^{-4}$

$f(F) := \mathrm{acalc} \cdot \frac{F}{(1 + \mathrm{acalc} \cdot F \cdot h)}$ $\qquad$ functional response (florets per second)

$F := 100 .. 20000$

$\frac{1}{h} = 0.823$ $\qquad$ asymptotic value functional response

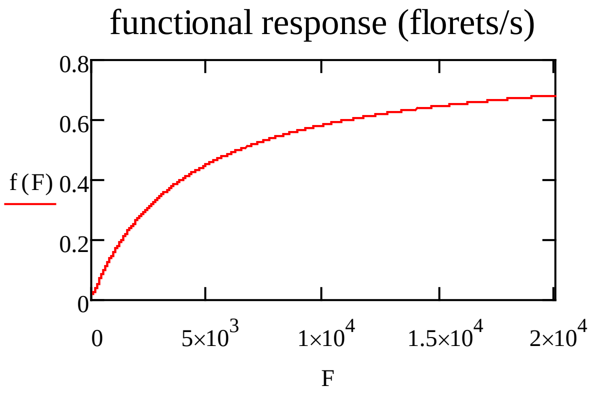

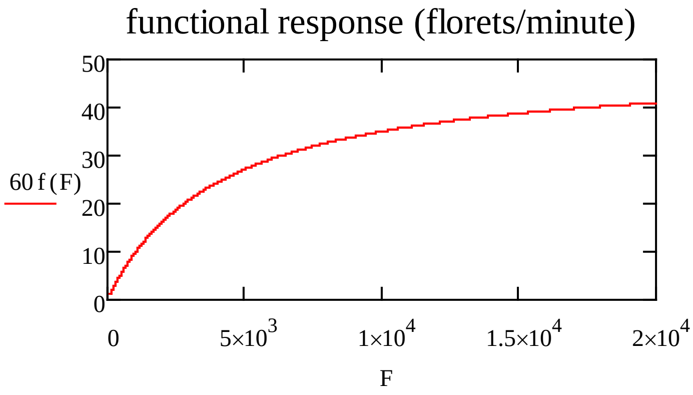

$f(2808) = 0.335$

Oil-seed rape

Own experiment 2015 (September)

Open flower density: 264 m^{-2}

Measured f (flowers per minute): 9 (+/- 2, n=3); 15 (+/- 2, n=5); average: 12.

Handling time per flower h (s): 4.1 (Free & Nuttall 1968)

MathCad code:

$flPerSec := \frac{12}{60}$ $flDensity := 264$ $h := 4.1$ handling time per flower (s)

$$GF(alpha) := (flPerSec) - \left(alpha \cdot \frac{flDensity}{1 + alpha \cdot flDensity \cdot h}\right)$$

$alpha := 0.0001$

$acalc := root(GF(alpha), alpha)$

$acalc = 4.209 \times 10^{-3}$

$f(F) := acalc \cdot \frac{F}{(1 + acalc \cdot F \cdot h)}$ functional response (flowers per second)

$F := 0..3000$

$\frac{1}{h} = 0.244$ asymptotic value functional response

functional response (flowers/s)

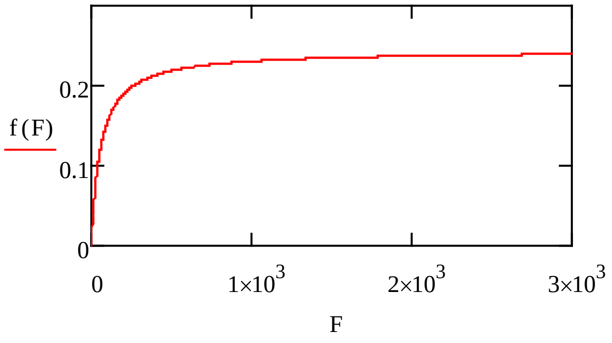

functional response (flowers/minute)

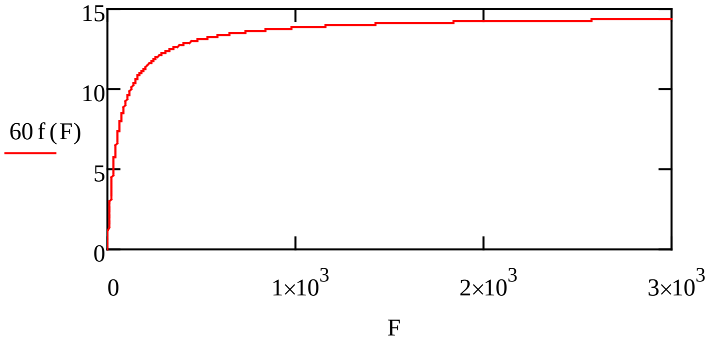

$f(flDensity) = 0.2$

References

Free JB, and Nuttall PM. 1968. The pollination of oilseed rape (Brassica napus) and the behaviour of bees on the crop. *The Journal of Agricultural Science* 71:91-94.

Goodwin RM, Cox HM, Taylor MA, Evans LJ, and McBrydie HM. 2011. Number of honey bee visits required to fully pollinate white clover (Trifolium repens) seed crops in Canterbury, New Zealand. *New Zealand Journal of Crop and Horticultural Science* 39:7-19.
