# Supplementary material for: An energetics-based honeybee nectar-foraging model used to assess the potential for landscape-level pesticide exposure dilution
Source: PeerJ. 2016 Aug 16;4:e2293. doi: 10.7717/peerj.2293 (PMC4991850; doi:10.7717/peerj.2293)
Supplement: Supplemental Information 2 [file peerj-04-2293-s002.pdf]

Supplementary Information – Recruitment Model

Title: An energetics-based honeybee nectar-foraging model used to assess the potential for landscape-level pesticide exposure dilution

Johannes M. Baveco, Alterra, Wageningen University & Research Center, Wageningen, The Netherlands

Andreas Focks, Alterra, Wageningen University & Research Center, Wageningen, The Netherlands

Dick Belgers, Alterra, Wageningen University & Research Center, Wageningen, The Netherlands

Jozef J.M. van der Steen, Plant Research International, Wageningen University & Research Center, Wageningen, The Netherlands

Jos J.T.I. Boesten, Alterra, Wageningen University & Research Center, Wageningen, The Netherlands

Ivo Roessink, Alterra, Wageningen University & Research Center, Wageningen, The Netherlands

Recruitment model

We implemented the differential equation model for the recruitment of foragers (Camazine & Sneyd 1991; Seeley et al. 1991), extended it to multiple resources (>= 2), and linked the coefficients to variables calculated in our foraging model.

The original model, figure 4 in Camazine & Sneyd 1991, was developed for two nectar sources A and B, and consisted of 7 compartments (p. 284):

1. H_a: unloading nectar from source A
2. H_b: unloading nectar from source B
3. D_a: dancing for nectar source A
4. D_b: dancing for nectar source B
5. A: foraging at nectar source A
6. B: foraging at nectar source B
7. F: following a dancer

Table 1. Parameter values, reproduced from Camazine & Sneyd 1991, table 2 (time values in minutes):

Parameter: definition	value
T_1: time from start of unloading to start of following, dancing, or foraging, A foragers	1.0
T_2: time from start of dancing to start of foraging, A foragers	1.5
T_3: time from start of foraging to start of unloading, A foragers	2.5
T_4: time from start of following dancers to start of foraging, A and B foragers	60
T_5: time from start of unloading to start of following, dancing, or foraging, B foragers	3.0
T_6: time from start of dancing to start of foraging, B foragers	2.0
T_7: time from start of foraging to start of unloading, B foragers	3.5
f_x^A: probability of abandoning A, per foraging trip	0.00
f_x^B: probability of abandoning B, per foraging trip	0.04
f_d^A: probability of dancing for A	1.00
f_d^B: probability of dancing for B	0.15

Equations, reproduced from Camazine & Sneyd 1991, Appendix:

$dA/dt = (1-f_x^A)(1-f_d^A)p_1H_a + p_2D_a + p_4f_l^AF-p_3A$

$dD_a/dt = f_d^A(1-f_x^A)p_1H_a – p_2D_a$

$dH_a/dt = p_3A – p_1H_a$

$dF/dt =f_x^Ap_1H_a + f_x^Bp_5H_b – p_4F$

$dB/dt = (1-f_d^B)(1-f_x^B)p_5H_b + p_6D_b + p_4f_l^BF-p_7B$

$dD_b/dt = f_d^B(1-f_x^B)p_5H_b – p_6D_b$

$dH_b/dt = p_7B – p_5H_b$

The rates p_1 to p_7 were obtained from $1/T_1$ to $1/T_7$. The recruitment rate of followers becoming foragers for A or B, f_l^A and f_l^B were defined as the likelihood of encountering a dancing dancer

$f_l^A = \tau_A D_a/(\tau_A D_a + \tau_B D_b)$ and $f_l^B = \tau_B D_b/(\tau_A D_a + \tau_B D_b)$

with τ_i representing the proportion of time at the dance floor spent in dancing. It was calculated as the product of the average number of dance circuits and the average circuit time, divided by the total time a bee is in compartment D_i (thus T_2 or T_6).

Our implementation produced the same behaviour as depicted in Camazine & Sneyd 1991 figure 4, when after 4 hours the sugar solutions in the feeders are switched (Fig. R1).

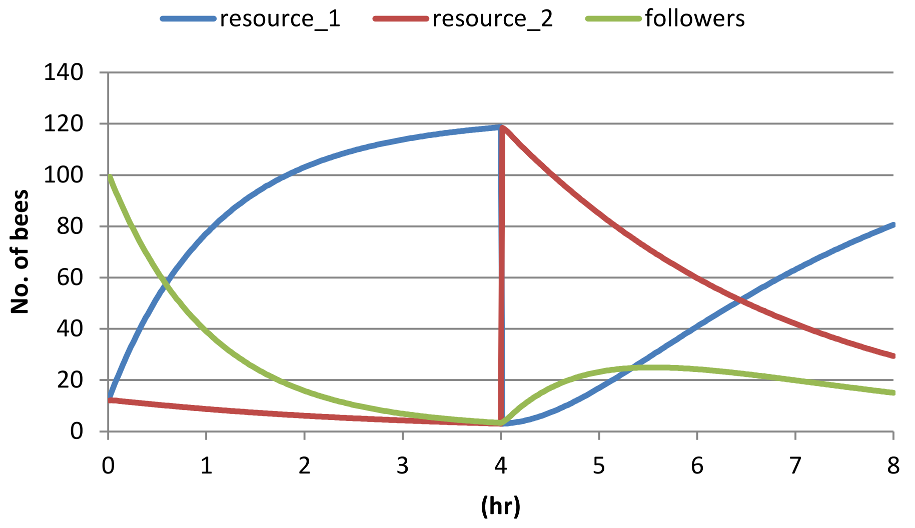

Figure R1. Number of bees foraging on each of the two feeders (dancers + unloaders + foragers at a source), and the number of dance followers.

Translating model coefficients

The coefficients listed above can be linked to the coefficients in the foraging model in the following way. The model of Camazine & Sneyd (1991) can also be simplified without modifying its behaviour by taking the compartments A and H_a (and B and H_b, etc) together and summing the times T_1 and T_3 to get the new rates.

Thus, $T_1 + T_3 = t_{trip} + t_{UD} - t_D$

In our foraging model we assume t_{UD} is constant. Simplifying the model of (Camazine & Sneyd 1991) we also assume that t_D is constant, and thus, implicitly, that t_U is constant. In reality t_U may decrease with increasing sugar content of the resource, while t_D may increase with resource quality. Because we are dealing mostly with attractive resources (high NEE) we

set $T_2 = t_D$ to 2 minutes, the maximum value for the most attractive resource in Camazine & Sneyd 1991. With a t_{UD} of 3 minutes, this implies that time unloading t_U is assumed to be 1 minute (the minimum value for the most attractive resource in Camazine & Sneyd (1991).

In our model NEE is assumed to define the attractiveness of a resource. Therefore we need to define f_x, the probability of abandoning the resource, and f_d, the probability of dancing for the resource, as functions of NEE: f_x(NEE) and f_d(NEE). To obtain values for f_x and f_d we need an estimate of NEE for the feeders in the experiment. Concentrations were 0.75 mol/L and 2.5 mol/L. With molecular weight of succrose 342 g, these are equivalent to 257 g/L and 856 g/L, and approximately 0.20 and 0.46 g sugar per g nectar.

For the feeders at 400 m distance energy expenditure *EE*, ignoring costs at the feeder, is thus (eq. 5) D * (e_U+e_L) / v = 400 * (0.037 + 0.075) / 4.17 = 10.7 J.

Energy intake *EI* (eq. 7) is then 0.20 * 17.2 * 32.5 = 111.8 J and 0.46 * 17.2 * 32.5 = 257.1 J.

NEE defined as (EI – EE) / EE becomes (111.8 – 10.7)/10.7=9.5 and (257.1 – 10.7)/10.7=23.0 for the 0.75 mol/L and 2.5 mol/L feeders, respectively.

We further have to define functions for the dependency of f_x and f_d on NEE, fitting to these two data points. For the probability to dance for a resource we assume a Hill function: $f_d(NEE) = \frac{NEE^p}{NEE^p + h^p}$ with exponent *p*=5 and *h* (the value for which f_d=0.5) set to 13 (see Fig. R2). For the probability to abandon a resource we assume an exponential function: $f_x(NEE) = e^{-a \cdot NEE}$ with *a*=0.325 (see Fig. R3). Clearly, with just two data points available, a linear relationship could be used as well, in particular for dancing probability (with truncation at 1). For abandoning probability we would in such case miss the likely steep increase with very low NEE.

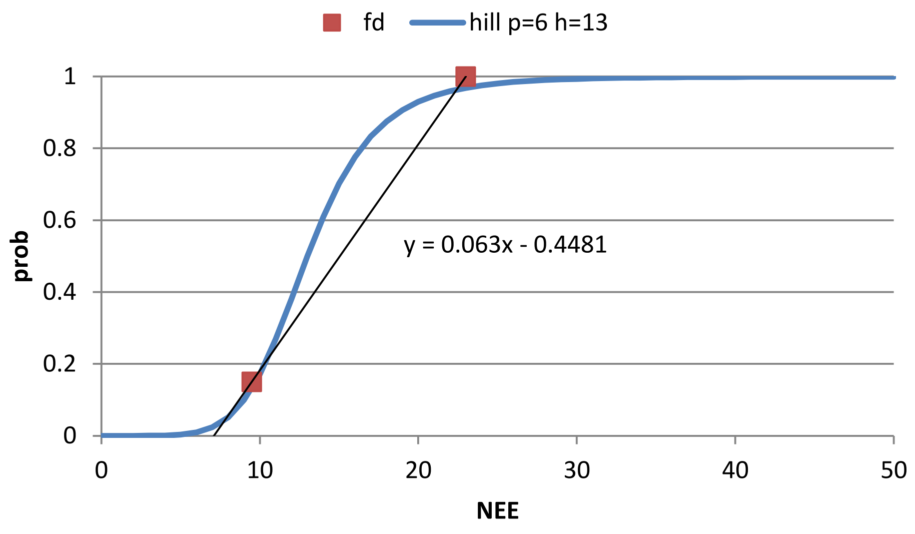

Figure R2. The Hill function used for the relationship between dancing probability and NEE.

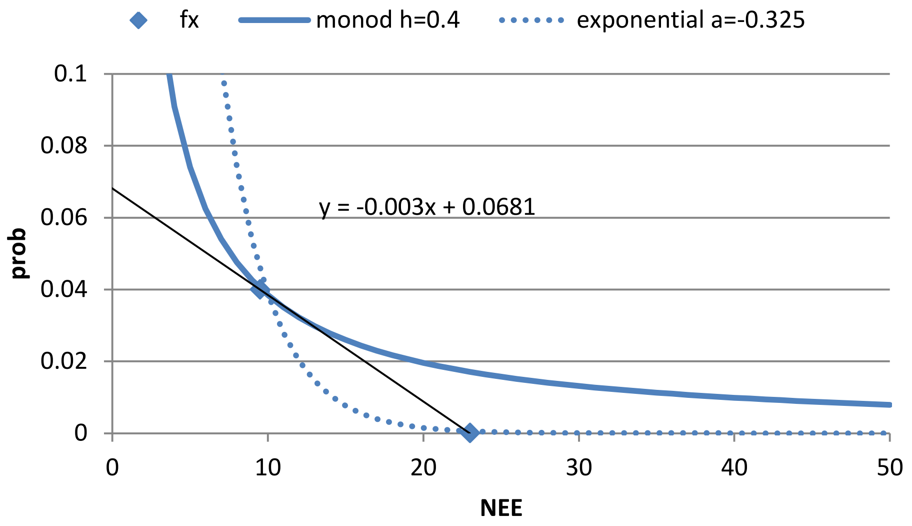

Figure R3. The exponential function used for the relationship between abandoning probability and NEE.

Camazine and Sneyd (1991) define τ_i as the proportion of time a bee in compartment D_i spends in dancing. This τ_i is the product of (average nr of circuits) and (average circuit time) divided by the time in D_i. The average nr of circuits N_{circ} depends on resource quality. In (Becher et al. 2014) is assumed, referring to (Seeley 1994) that

$$N_{circ}(NEE) = 1.16 * NEE$$

with a maximum value of 117 circuits (referring to (Seeley & Towne 1992)).

When average circuit time and time in D_i are assumed to be constant, τ_i scales linearly with the number of circuits danced, and it is simpler to formulate it as:

$$\tau_i(NEE) = \frac{\min(1.16 \cdot NEE, 117)}{117}$$

With the relationships and coefficients as described above (unchanged T_1, T_3 and T_2=T_6=2.0) the implementation based on NEE produces again (as should be expected) in the same dynamics (Fig. R4).

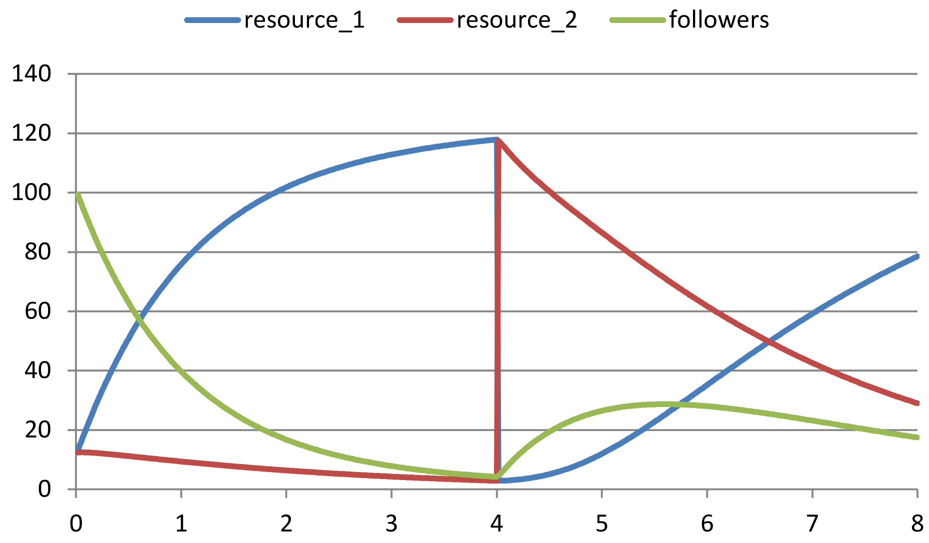

Figure R4. The NEE-based implementation of the Camazine and Sneyd (1991) model produces identical behaviour.

Dynamics over multiple hours

We applied the model over multiple hours. Each hour was a separate run, with the initial number of followers set to 100 (this was varied in a simple sensitivity analysis), and with the initial number of dancers (D) and foragers (F here representing foraging & unloading bees) copied from the final state of the previous run (if the resource was also present in the previous run). The relative values of bees exploiting resource i given by $r_i = D_i + F_i / \Sigma(D_j + F_j)$ at the end of each run were used in the foraging model as the number of active foragers exploiting each resource (so r_i multiplied by the assumed number of active foragers in this same hour). Figures R5 and R6 show how this could work out for r_i in an actual simulation of the foraging model.

The constant number of followers at the start of each hour defines a kind of turn-over rate for foragers, with larger values speeding up the dynamics and thereby increasing the differences between resources faster. Non-constant numbers could be used, when data are available on the actual fluctuating numbers in a real hive. Comparison of the results for all scenarios with the number of followers set to 50 and to 200 instead of 100 showed that the impact of this parameter was small (Fig. S12), with slightly less patches exploited over the day when the parameter was large and the process of focussing on the best patches faster (Fig. S13)

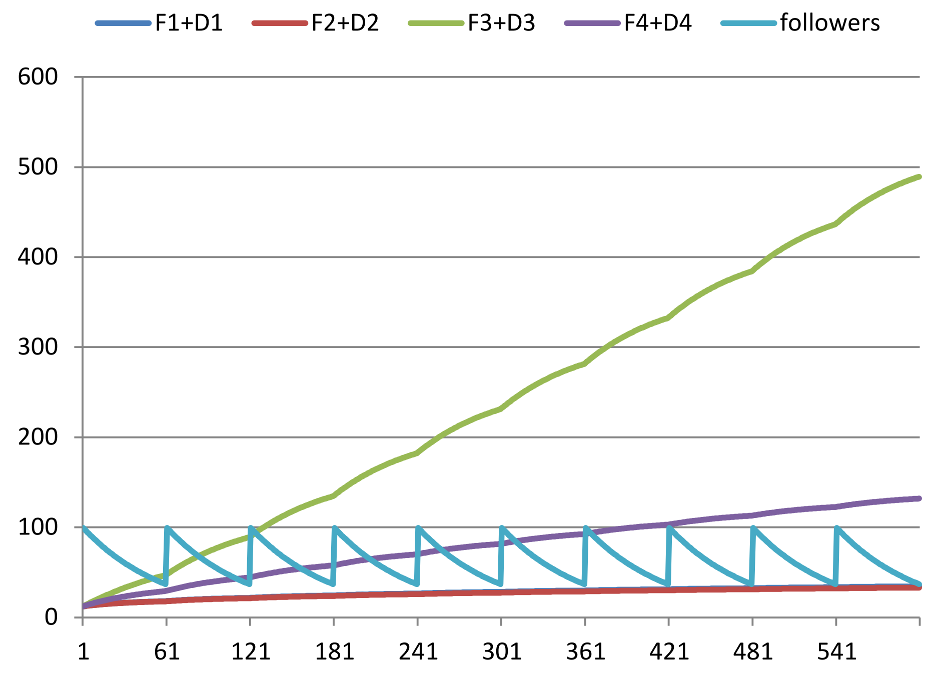

Figure R5. The numbers (F+D) of bees exploiting a resource, in case of 4 resources, as simulated by the recruitment model.

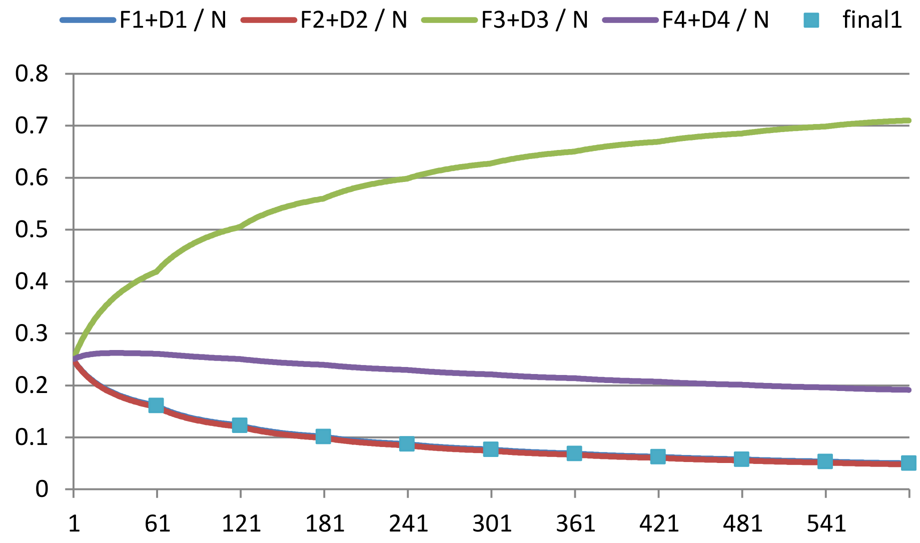

Figure R6. The fraction of the active foragers pool dedicated to each of 4 resources (values as in figure R5) with the dots (only displayed for resource 2) representing the values that are actually used in the foraging simulation. With this approach the same number of active foragers each hour is used as in the "single-optimal" version of the model.

Futher assumptions

The minimum NEE for resources to be considered was set to 20, implying that energetic net profit (gain – cost) had to be 20 times the cost. For the “Alternative Fields” scenarios lower values worked well. However, for the other two scenarios, where numerous high quality field margins or off-field habitats could be present, this led to too large and unrealistic numbers of resources being considered in the recruitment model. Therefore a minimum NEE was set to 20 (as is also an option in the BEEHAVE model (Becher et al. 2014)).

References

Becher MA, Grimm V, Thorbek P, Horn J, Kennedy PJ, and Osborne JL. 2014. BEEHAVE: a systems model of honeybee colony dynamics and foraging to explore multifactorial causes of colony failure. *Journal of Applied Ecology* 51:470-482.

Camazine S, and Sneyd J. 1991. A MODEL OF COLLECTIVE NECTAR SOURCE SELECTION BY HONEY-BEES - SELF-ORGANIZATION THROUGH SIMPLE RULES. *Journal of Theoretical Biology* 149:547-571.

Seeley TD. 1994. HONEY-BEE FORAGERS AS SENSORY UNITS OF THEIR COLONIES. *Behavioral Ecology and Sociobiology* 34:51-62.

Seeley TD, Camazine S, and Sneyd J. 1991. COLLECTIVE DECISION-MAKING IN HONEY-BEES - HOW COLONIES CHOOSE AMONG NECTAR SOURCES. *Behavioral Ecology and Sociobiology* 28:277-290.

Seeley TD, and Towne WF. 1992. TACTICS OF DANCE CHOICE IN HONEY-BEES - DO FORAGERS COMPARE DANCES. *Behavioral Ecology and Sociobiology* 30:59-69.
