# Supplementary material for: An energetics-based honeybee nectar-foraging model used to assess the potential for landscape-level pesticide exposure dilution
Source: PeerJ. 2016 Aug 16;4:e2293. doi: 10.7717/peerj.2293 (PMC4991850; doi:10.7717/peerj.2293)
Supplement: Supplemental Information 3 [file peerj-04-2293-s003.docx]

Download the Squeak Smalltalk program (MSWindows version) containing the model code from:

<http://webdocs.alterra.wur.nl/internet/landschap/EMM/BeeRisk/BeeRisk.zip>

Unzip it (beerisk directory is part of the zipped data). The created \beerisk directory has a subdirectory \beerisk\ecotalk that contains the 'model scripts' specifying the models and their settings, for each of the scenarios.

Download the required datasets (mainly spatial data in asciigrid or ESRI shape-file format) from:

<http://webdocs.alterra.wur.nl/internet/landschap/EMM/BeeRisk/ETInput.zip>

Unzip ETInput.zip in the new beerisk directory, resulting in a subdirectory \beerisk\ETInput containing subdirectories with input data for each model version.

Thus the result of both actions should be a directory

\beerisk

with subdirectories

\beerisk\ecotalk

\beerisk\ETInput

In the beerisk directory, double-click the executable Squeak.exe. This will automatically launch the BeeRisk.image.

If this isn't working, the BeeRisk.image can be 'thrown on' the executable, or a batch file created containing the line:

d:\BeeRisk\squeak.exe d:\BeeRisk\BeeRisk.image

(with paths adapted)

The BeeRisk.image and BeeRisk.changes files belong together and should not be changed nor edited by hand. For non-windows OS the same (image and changes) files can be used with executables and libraries that can be downloaded from <http://Squeak.org>
