# Supplementary material for: An energetics-based honeybee nectar-foraging model used to assess the potential for landscape-level pesticide exposure dilution
Source: PeerJ. 2016 Aug 16;4:e2293. doi: 10.7717/peerj.2293 (PMC4991850; doi:10.7717/peerj.2293)
Supplement: Supplemental Information 4 [file peerj-04-2293-s004.pdf]

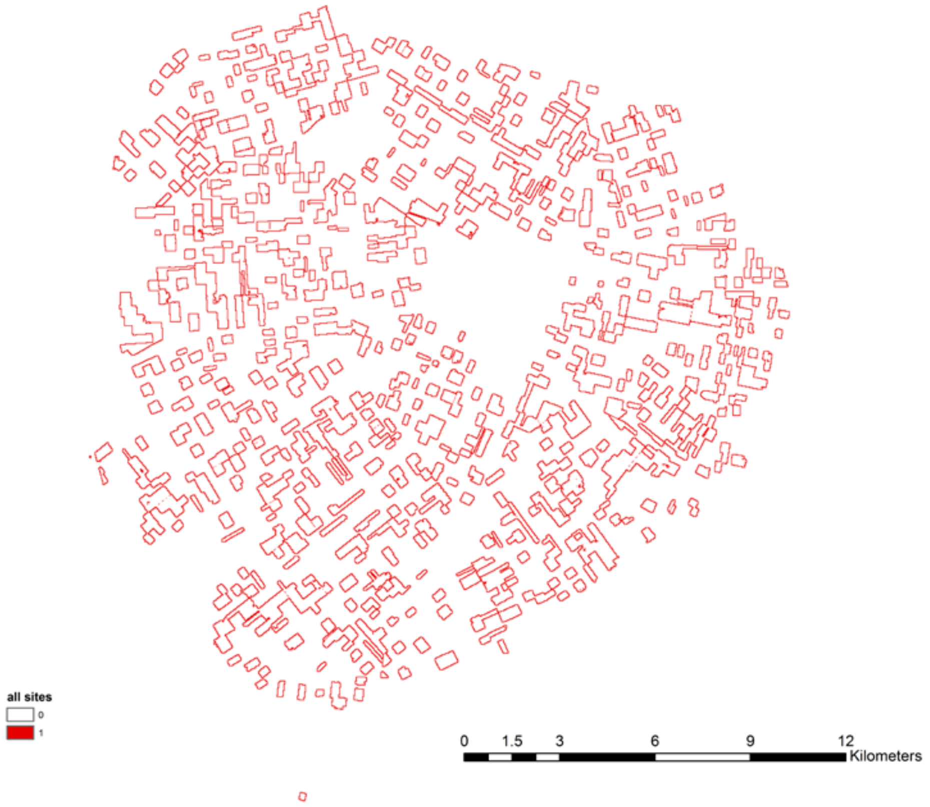

Figure S1A. All potential beehive locations (N=48854) in the landscape (cells bordering OSR fields in a raster with 25 m resolution, see Fig. 1).

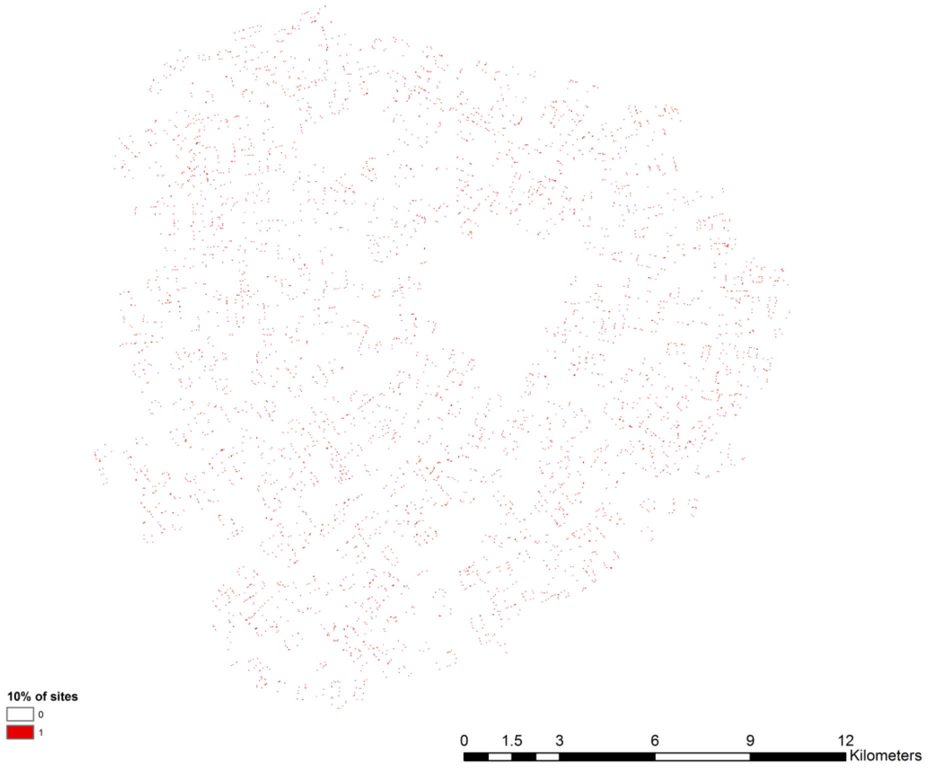

Figure S1b. A random selection of approx. 10 % of all potential beehive locations, as used in the scenario simulations (N=4931).

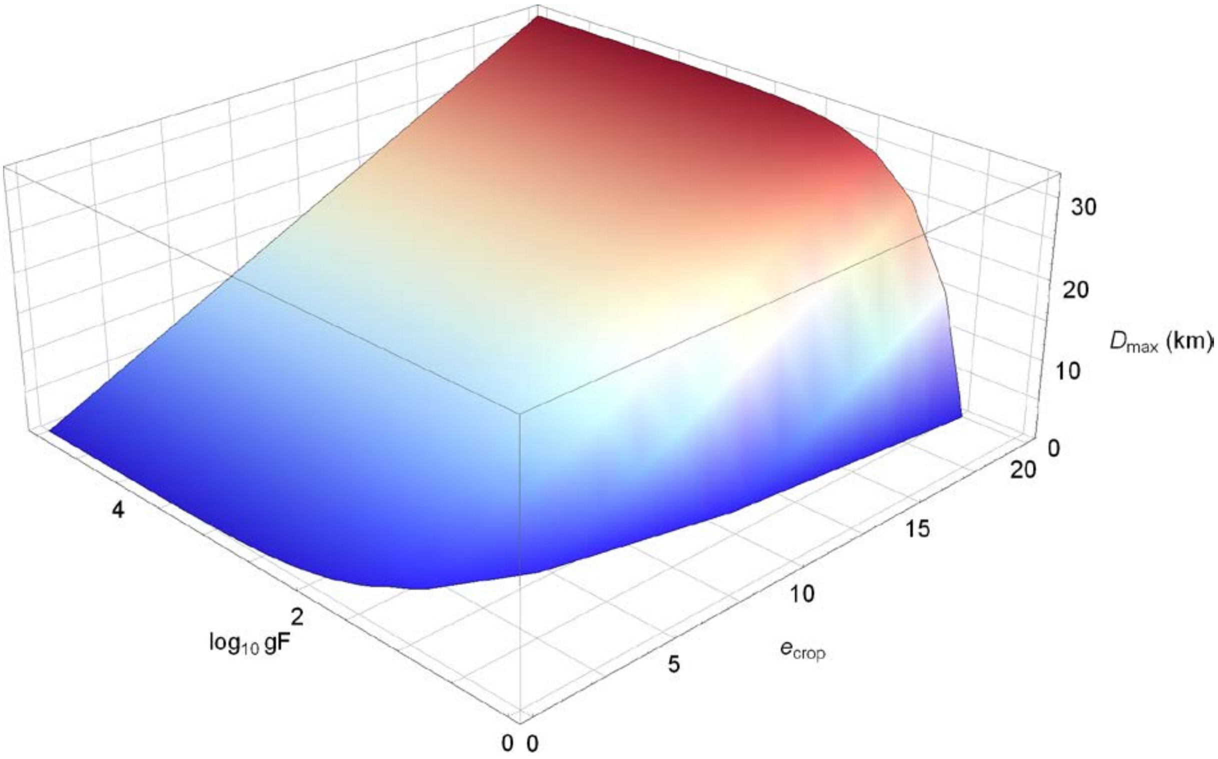

Figure S2. Maximum distance increases linearly with energy content of the resource (e_R here referred to as e_{crop}, in J mg^{-1}) and asymptotically with resource density gF (mg nectar m^{-2}) or its ^{10}log as shown here.

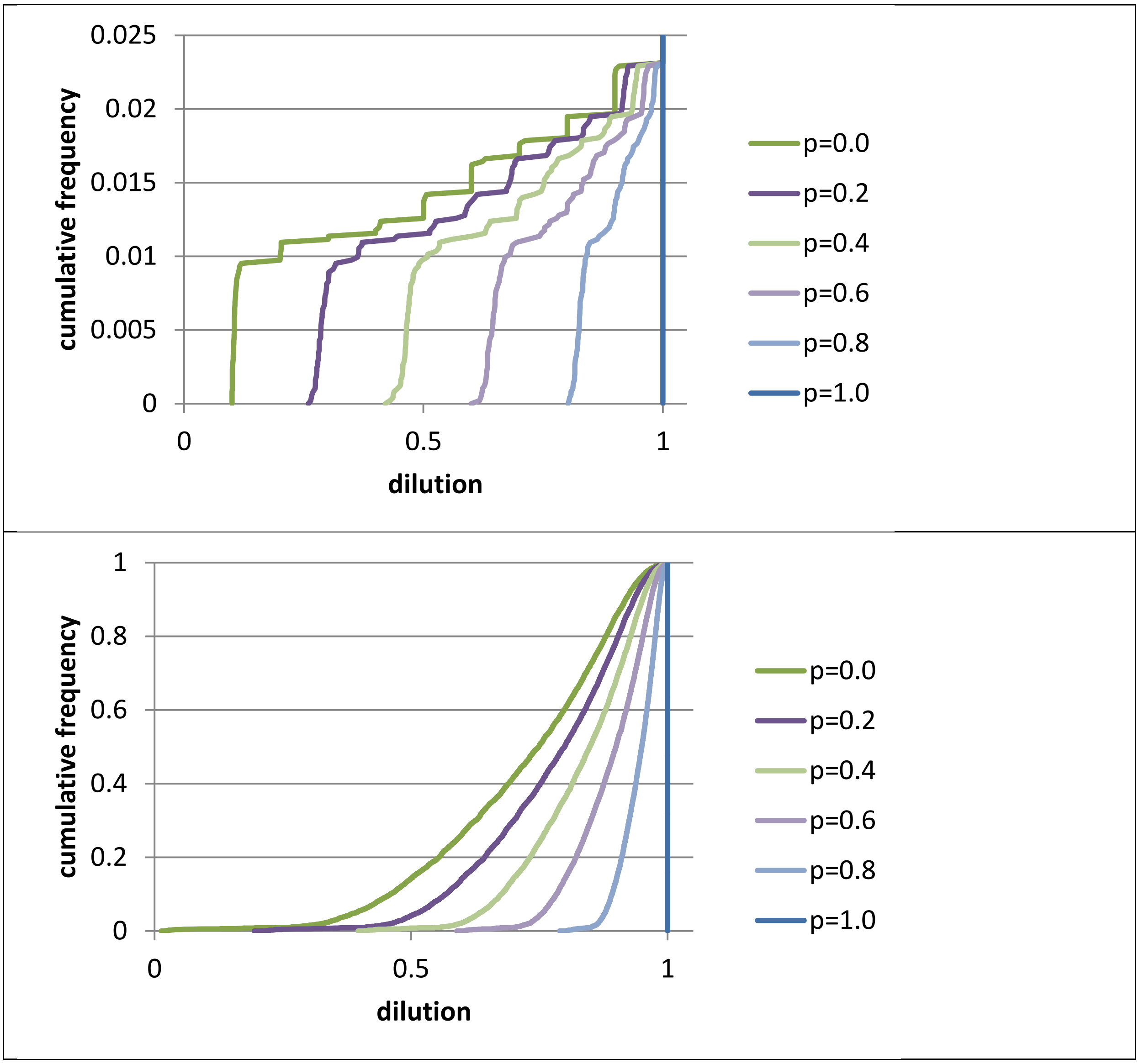

Figure S3. Cumulative frequency distribution of dilution factors for all sites. Alternative Fields scenario with only the target crop, but not all fields being treated. Top: SO-model. Fewer than 2.5 % of the 4931 sites have a dilution factor < 1. For these sites, the probability of target crop spraying *p* determines the dilution that can be obtained. Bottom: RL-model. All sites have a dilution factor < 1, but the frequency of sites with higher dilution (dilution factor approaching 0) drops quickly to zero.

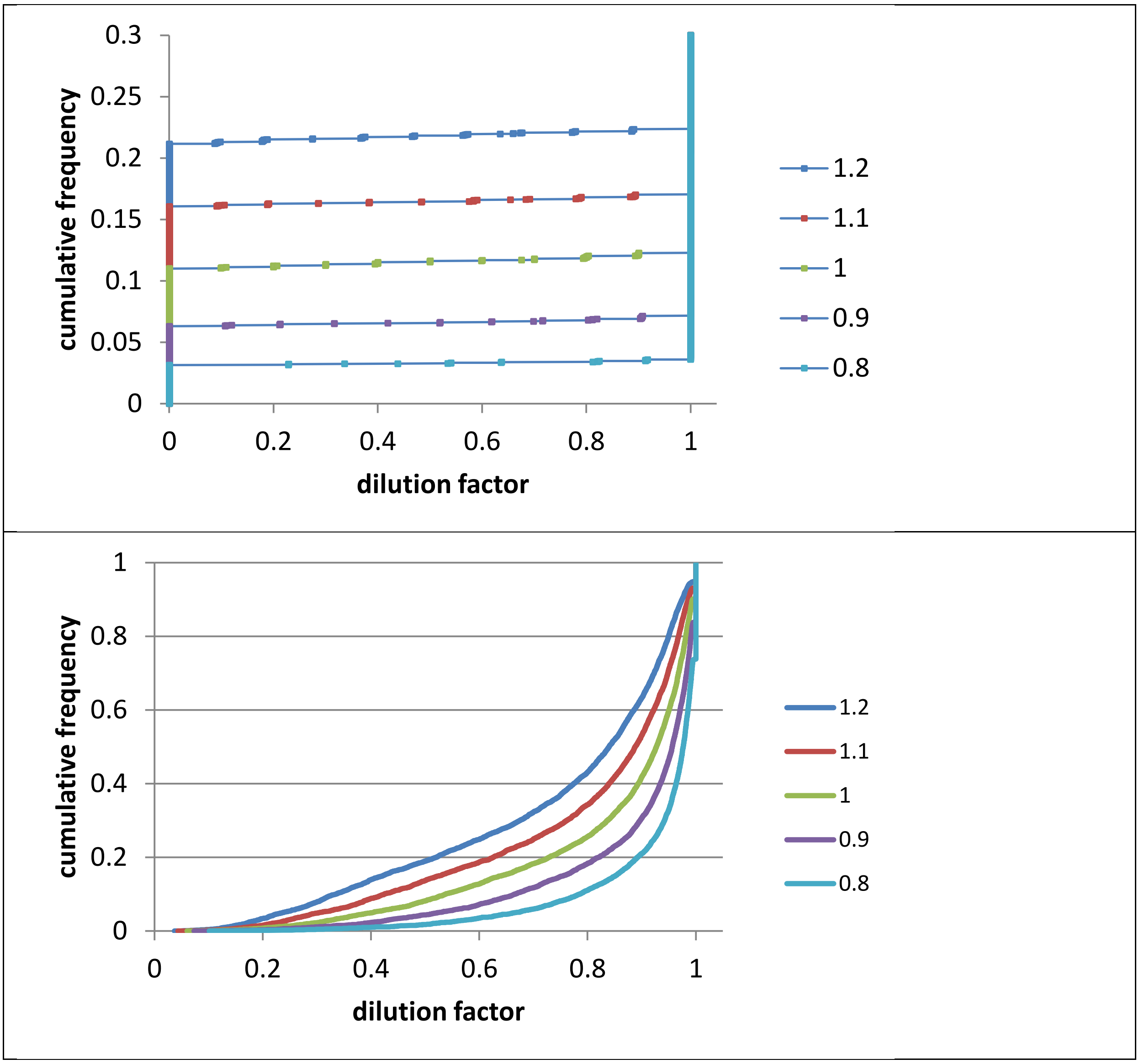

Figure S4. Cumulative frequency of dilution factors for range of values of relative sugar content of alternative resource. Alternative Fields scenario with a second (similar) crop being present with the same characteristics as OSR, but different sugar content. Multiplication factor for sugar content relative to OSR 0.8, 0.9, 1, 1.1 and 1.2. Top: SO-model; bottom: RL-model.

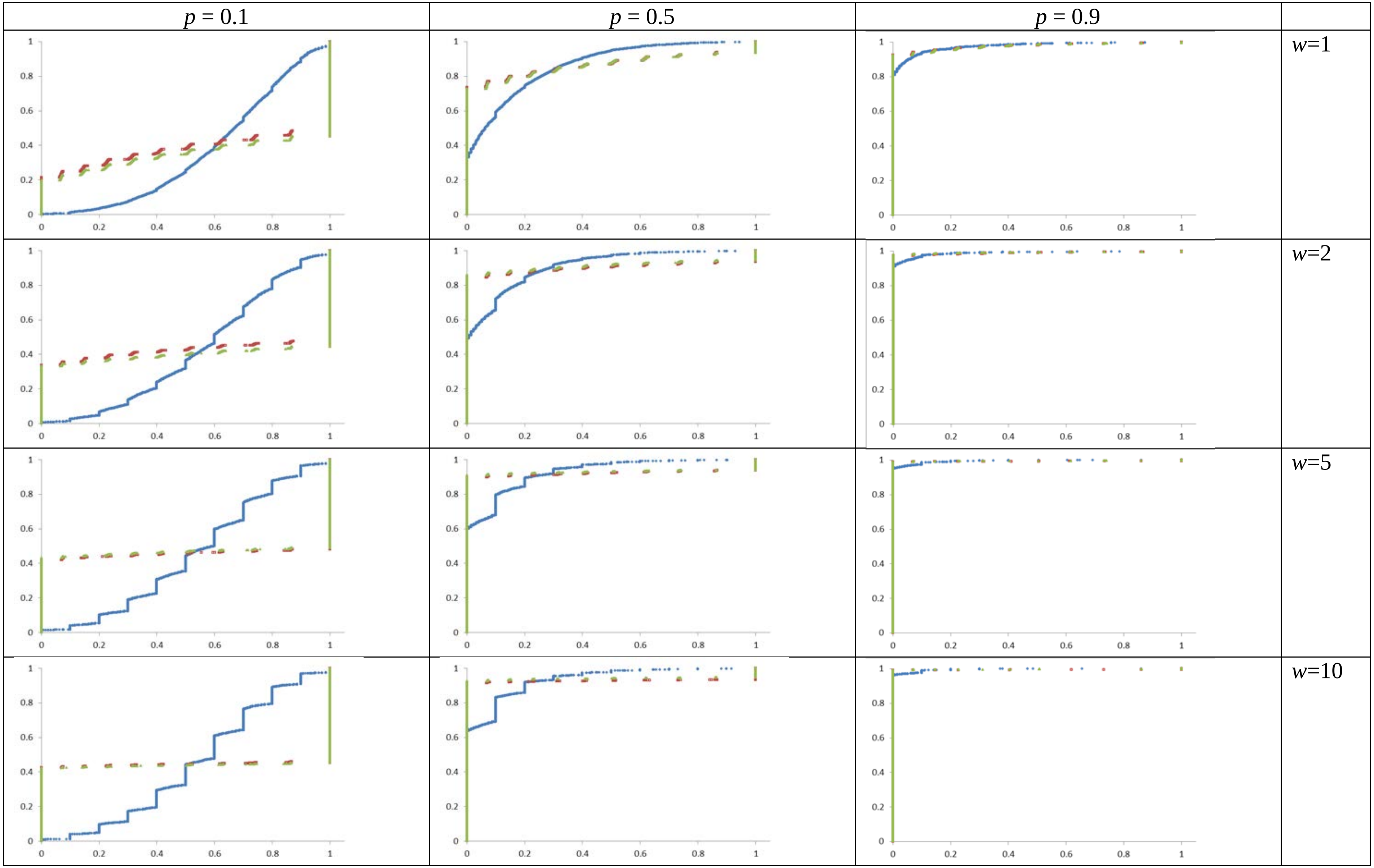
p = 0.1
p = 0.5
p = 0.9
w=1
w=2
w=5
w=10

	p = 0.1	p = 0.5	p = 0.9
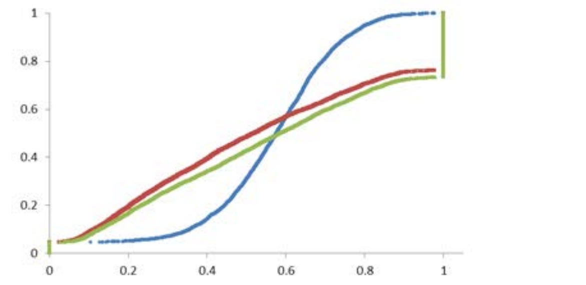	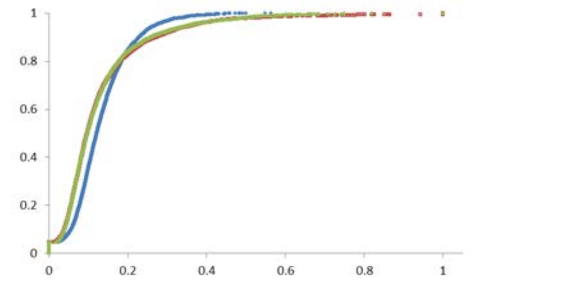	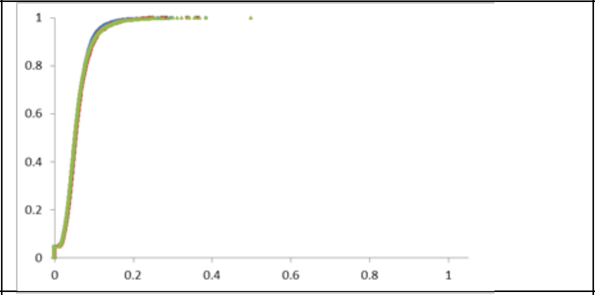	w=1
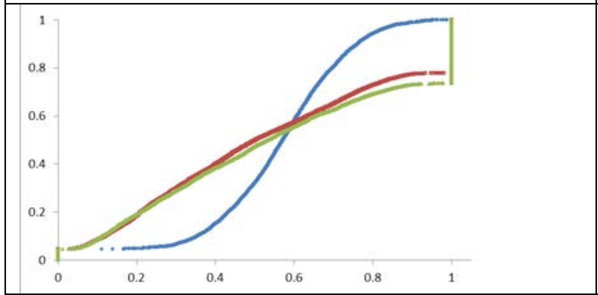	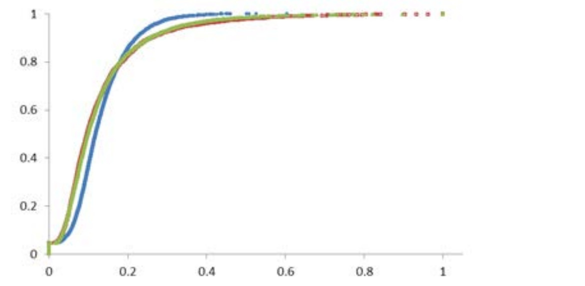	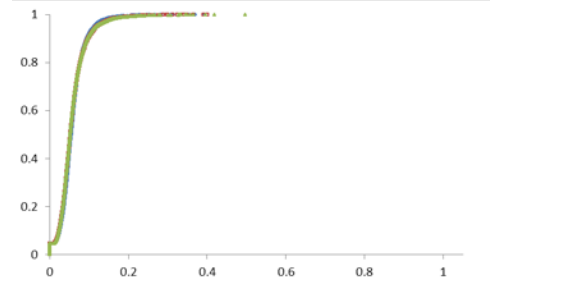	w=2
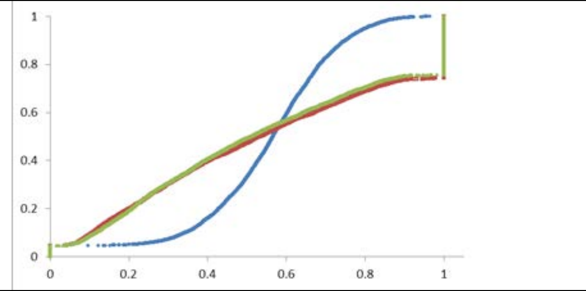	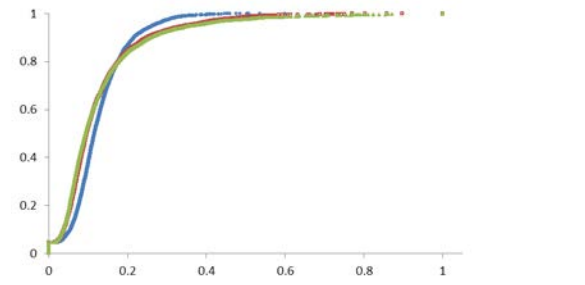	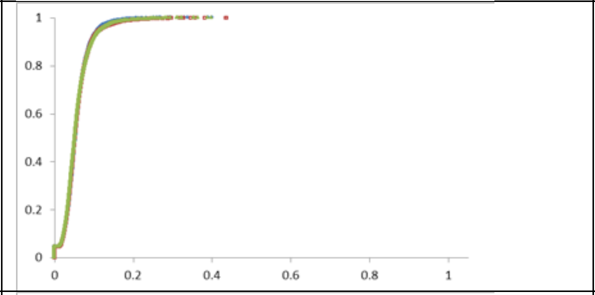	w=5
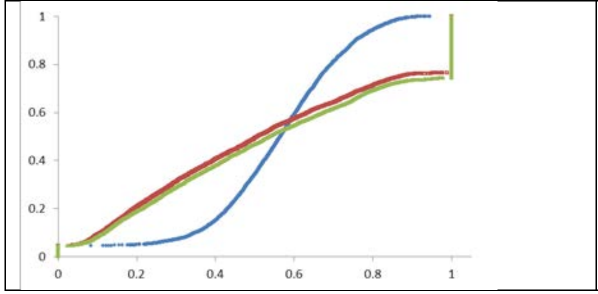	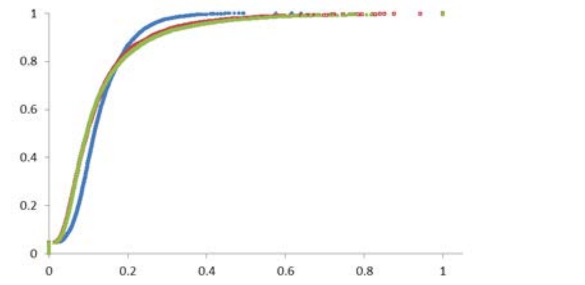	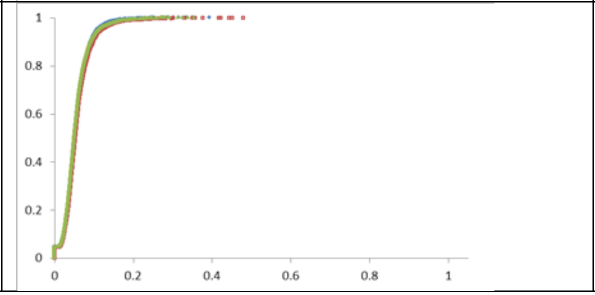	w=10

Figure S5. Flower Strips scenario. Some of the cumulative distributions of dilution factors, for different combinations of the probability of a flower strip being present on one the four sides of a field (p) and width (w) of the flower strip, in meters. Blue line: the cumulative distribution resulting when for each site the results are averaged over 10 simulations (each simulation producing a different landscape configuration in terms of flower strips present). First page: SO-model; second page: RL-model.

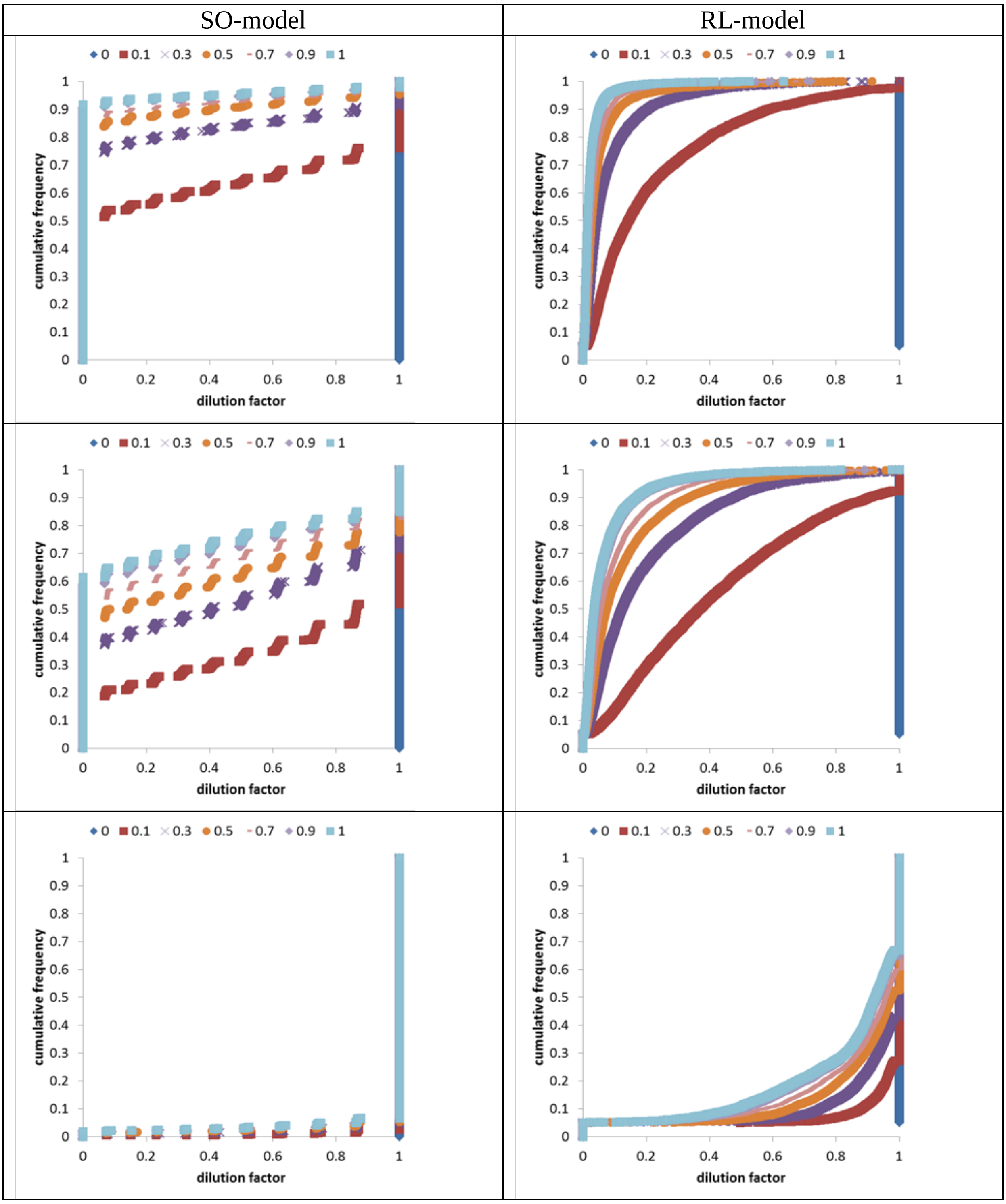

Figure S6. Cumulative frequency distribution of dilution factors, for different values of the probability of an off-field patch being a resource patch. Upper row: high flower density on off-field patches; middle row: intermediate flower density; bottom row: low flower density. Left column: SO-model; right column: RL-model. Results from single simulations (variation between simulations was small).

SO-model default | RL-model default

“Alternative Fields”

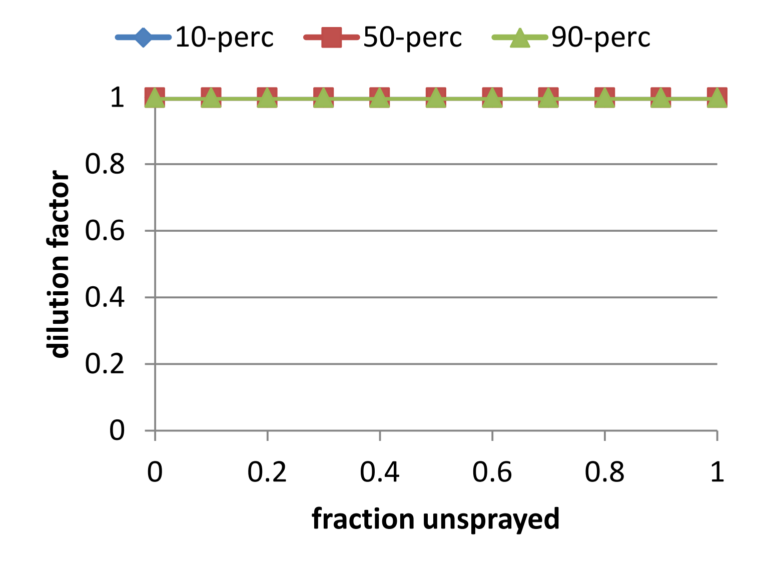

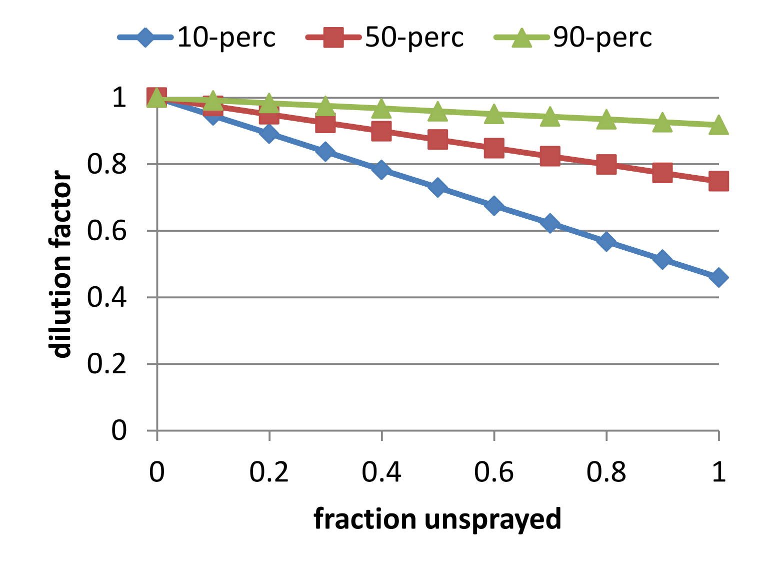

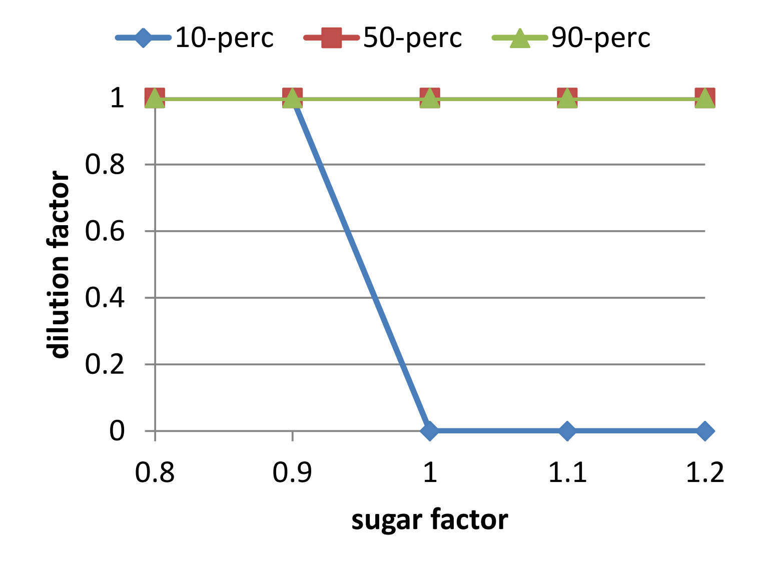

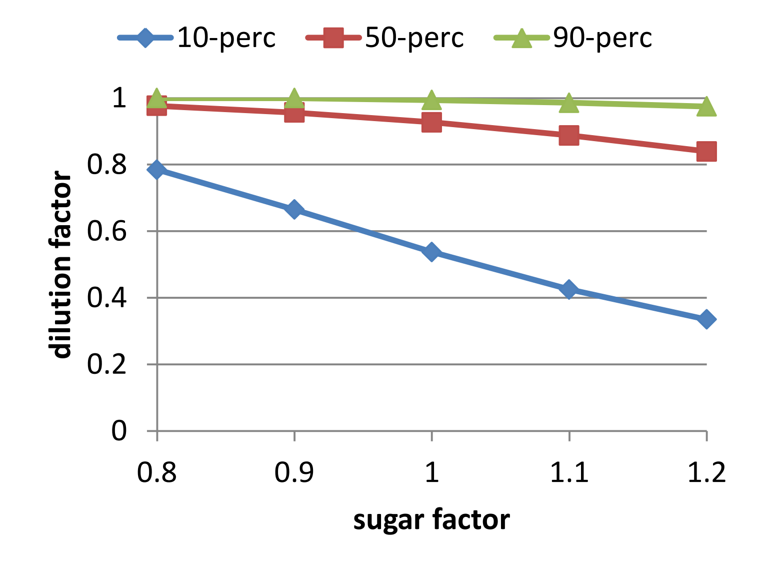

“Flower Strips” 1, 2, 5 and 10 m wide

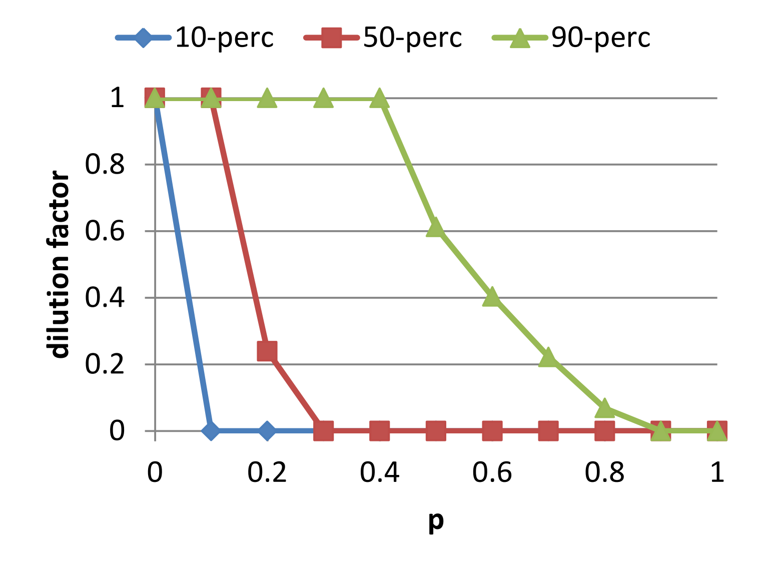

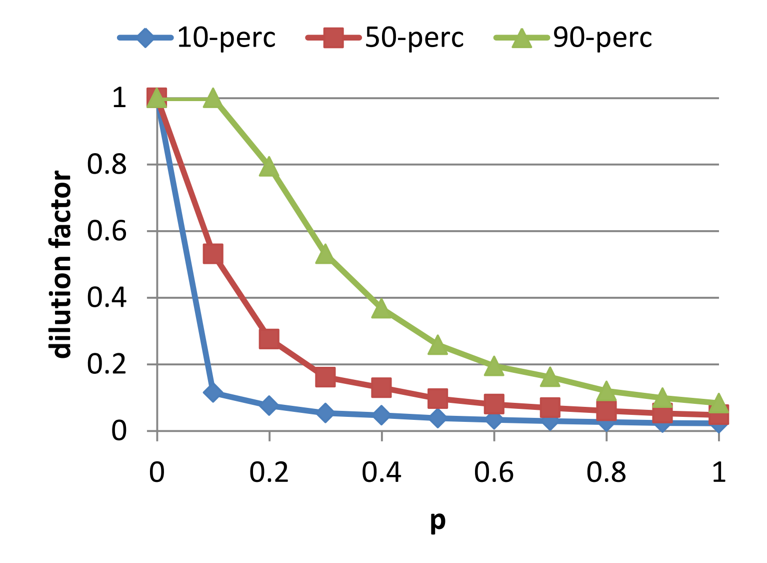

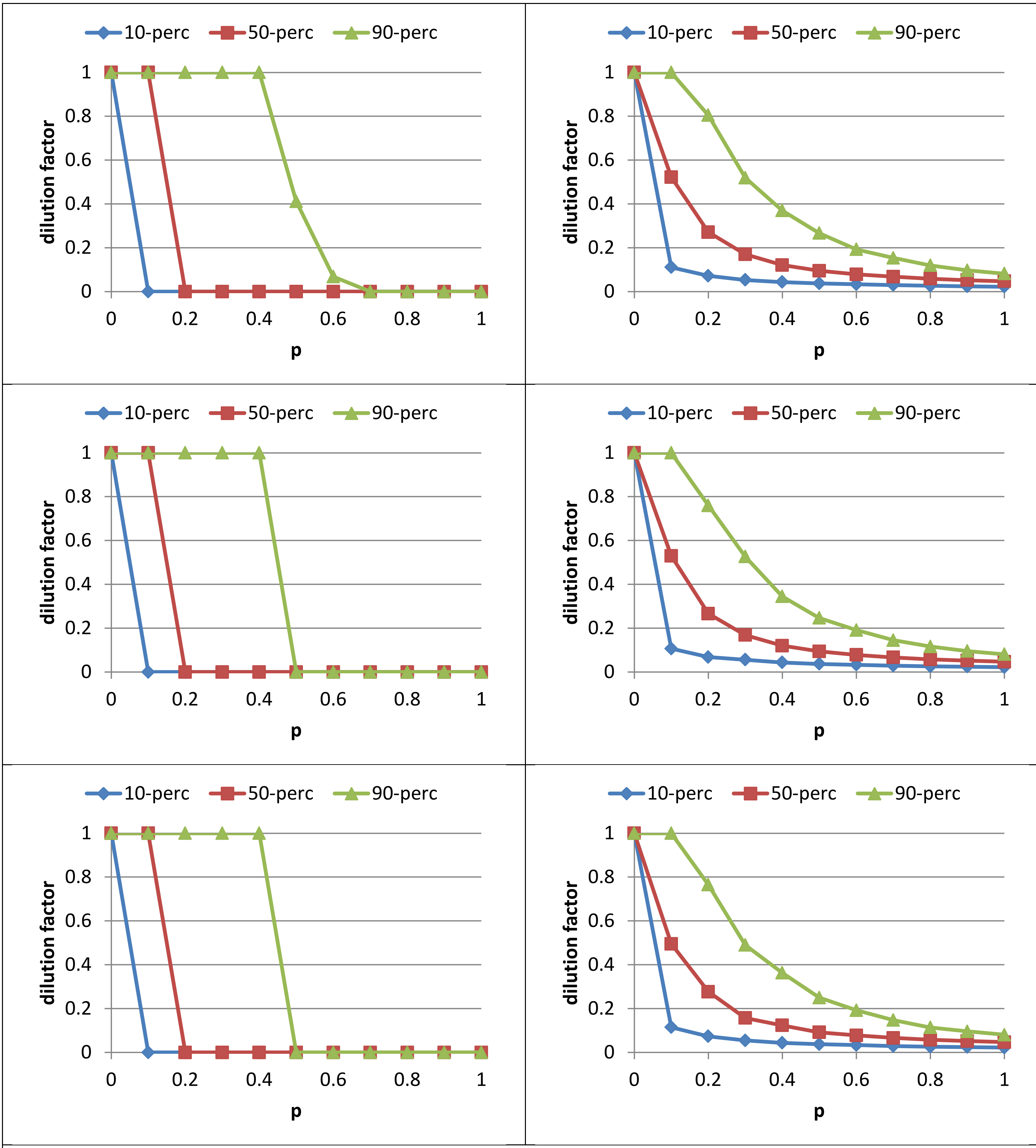

"Off-field Habitats" high, medium and low quality

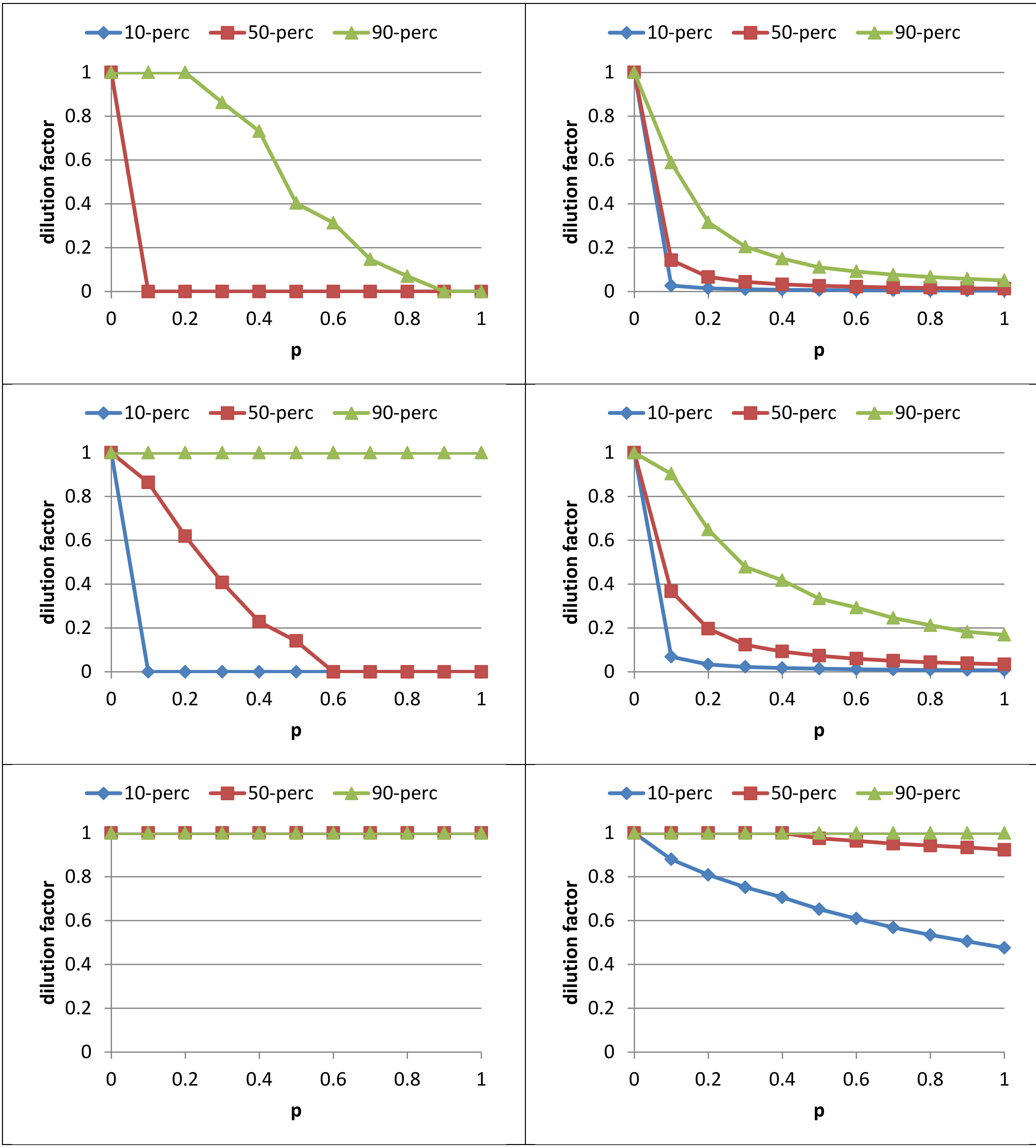
10-perc
50-perc
90-perc
dilution factor
p
0
0.2
0.4
0.6
0.8
1

Figure S7. The 10-, 50- and 90-percentile dilution factors for all scenarios, for the SO-model (left) and the RL-model (right). Rows from top to bottom: “alternative fields” (2x), “flower strips” (1, 2, 5, 10 m width), “off-field habitats” (high, medium, low quality).

SO-model default	RL-model default

“Alternative Fields”

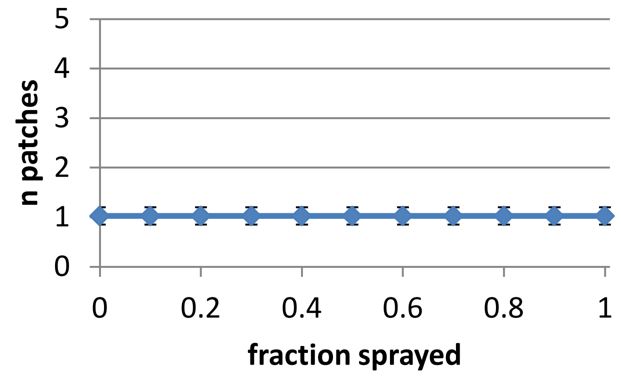

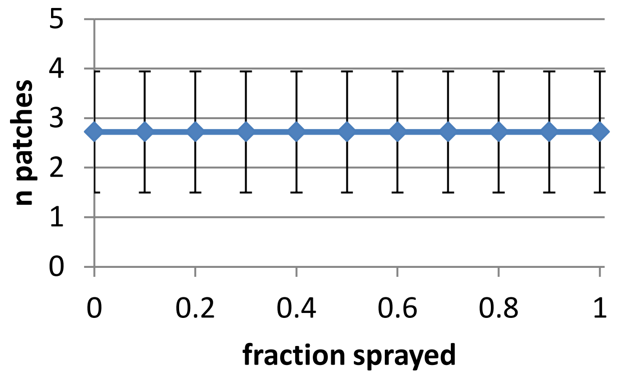

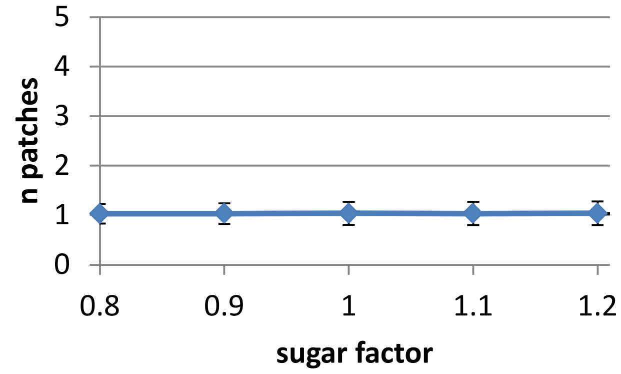

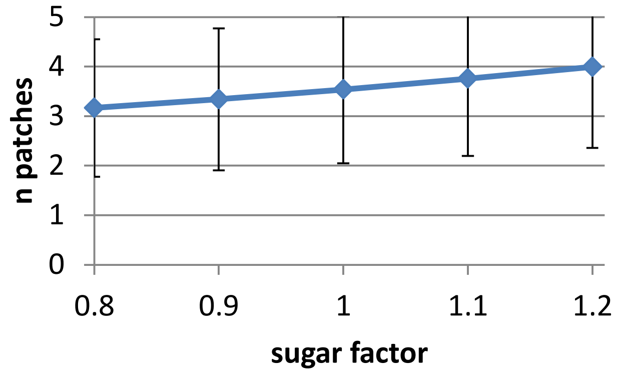

“Flower Strips” 1, 2, 5 and 10 m wide

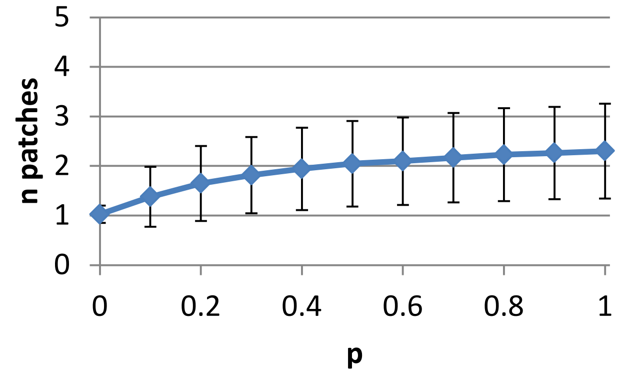

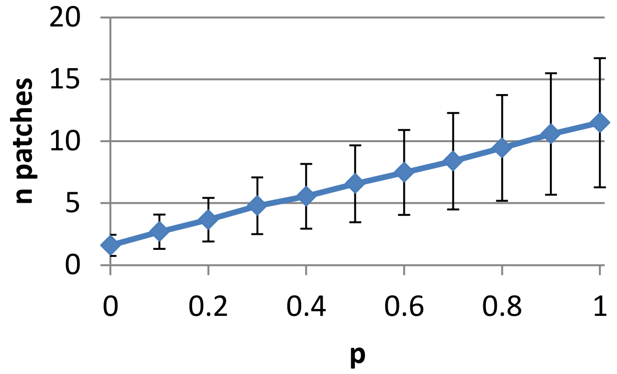

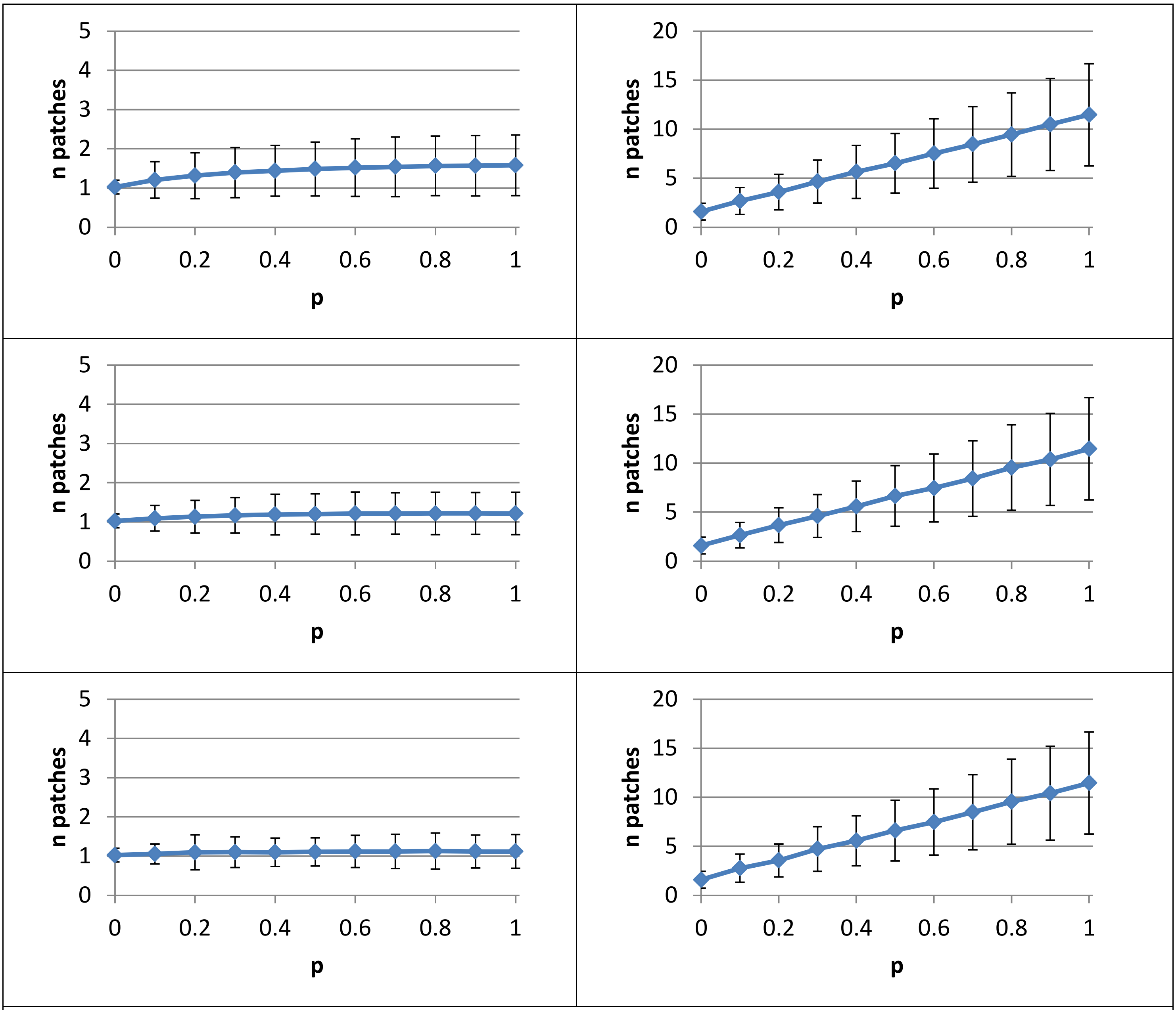

“Off-field Habitats” high, medium and low quality

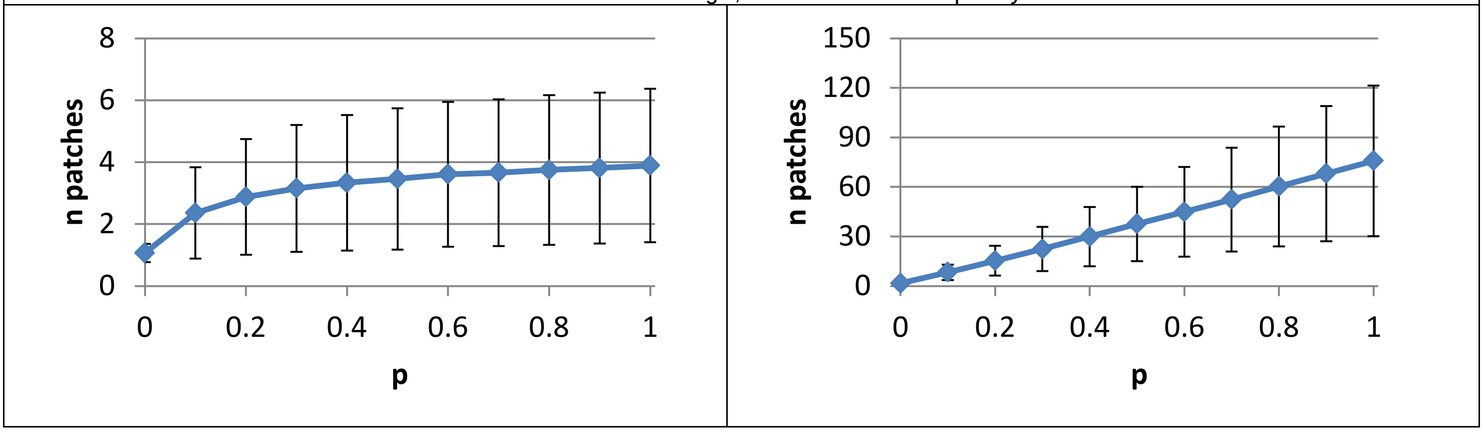

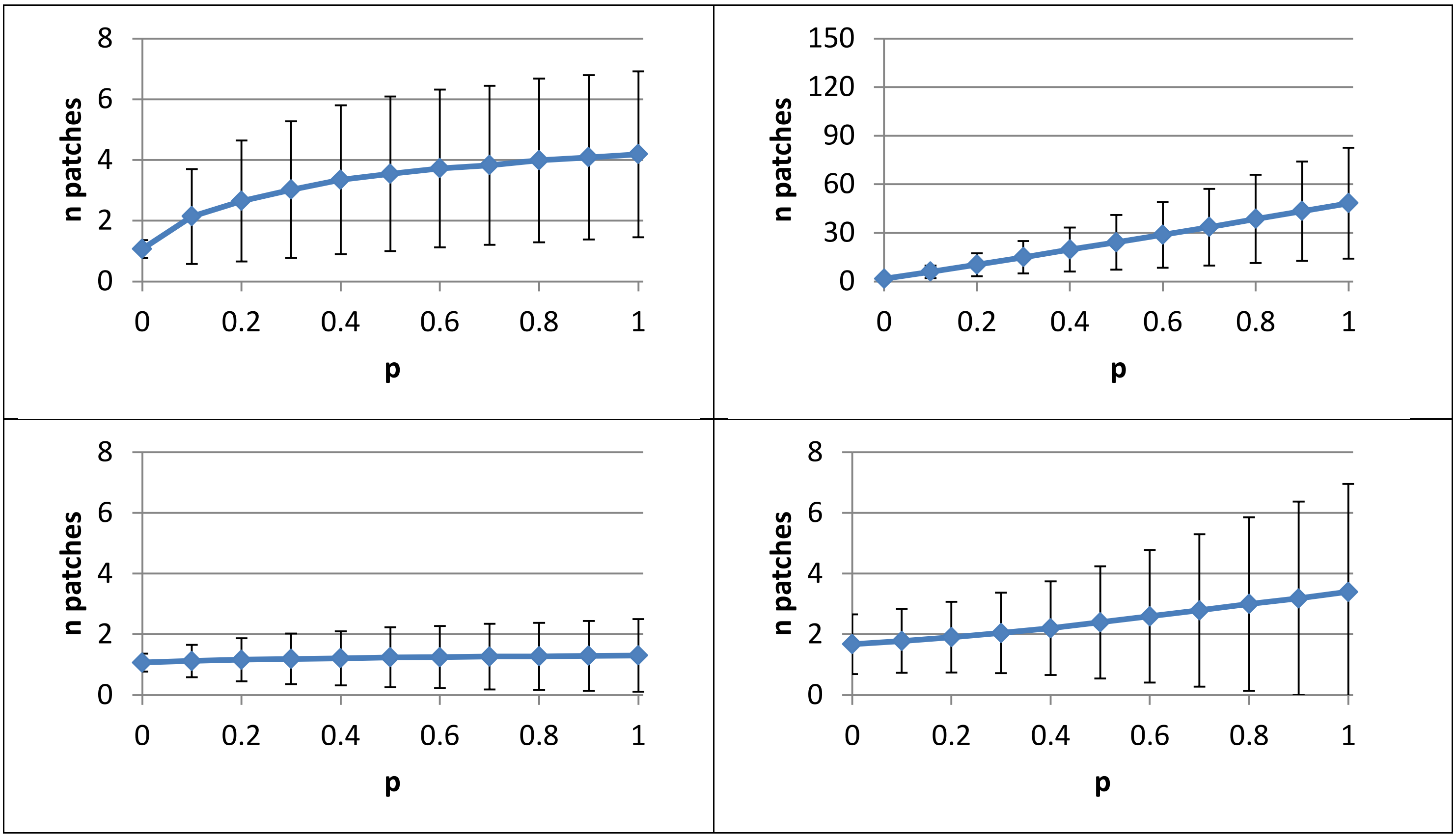

Figure S8. Number of patches exploited over the day in all scenarios, for the SO-model (left) and the RL-model (right). Rows from top to bottom: “alternative fields” (2x), “flower strips” (1, 2, 5, 10 m width), “off-field habitats” (high, medium, low quality).

SO-model	RL-model
“Flower Strips” 1m	

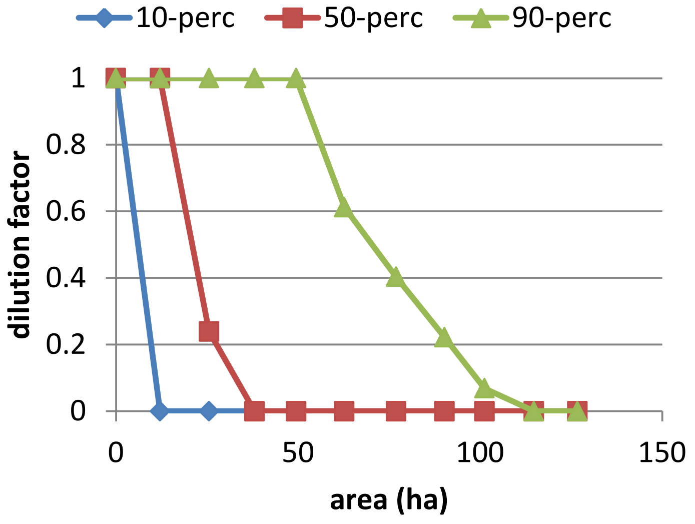

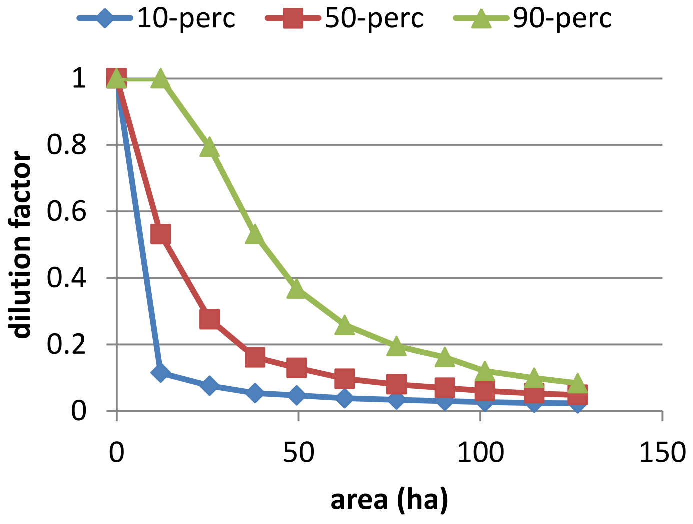

“Flower Strips” 2m

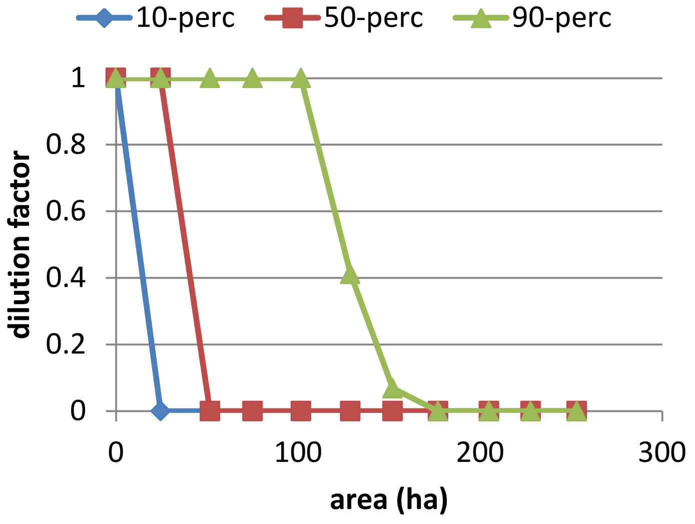

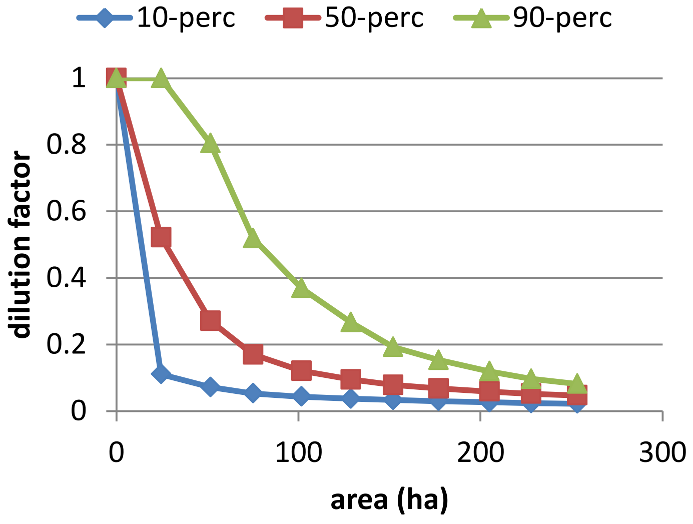

“Flower Strips” 5m

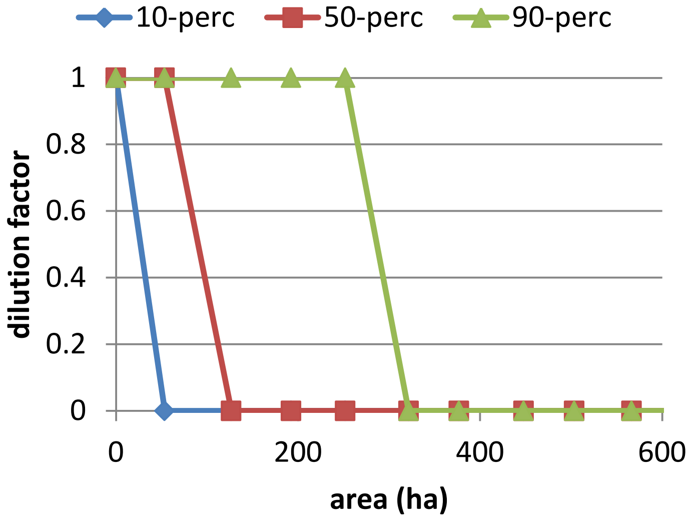

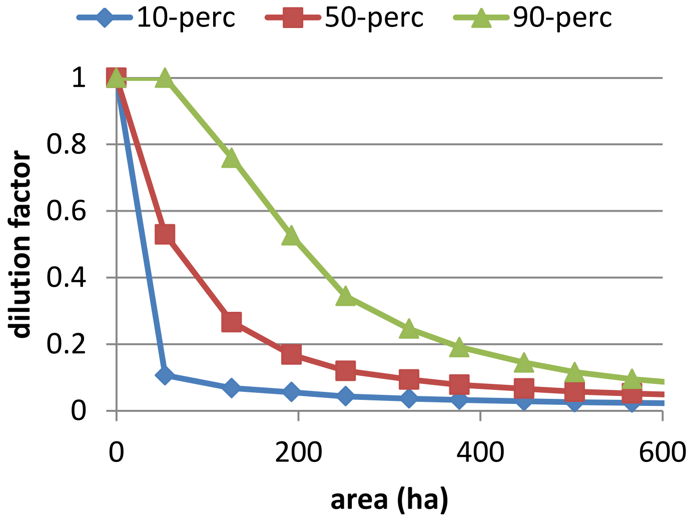

“Flower Strips” 10m

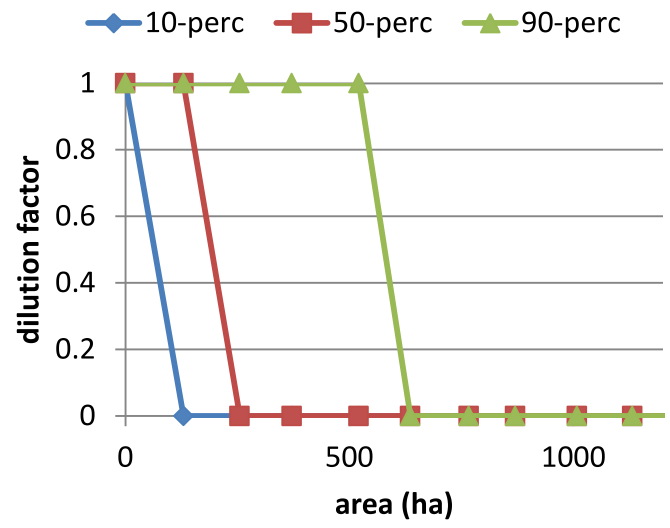

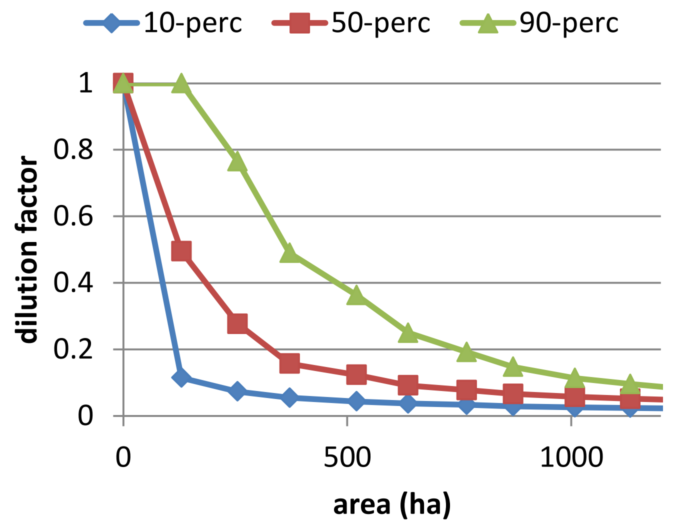

“Off-field Habitats” high quality

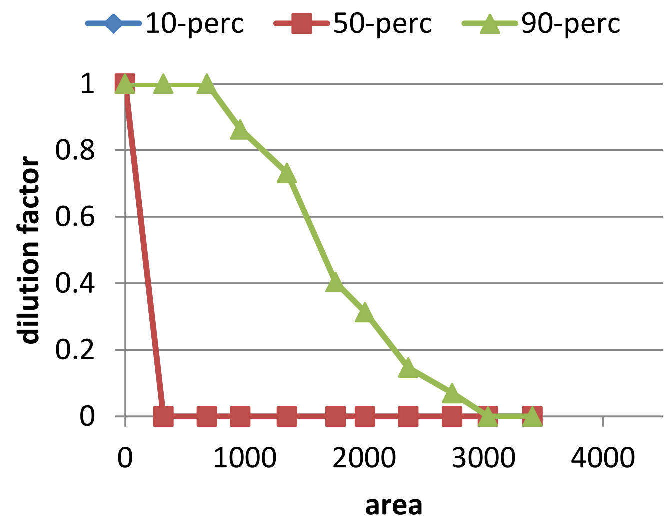

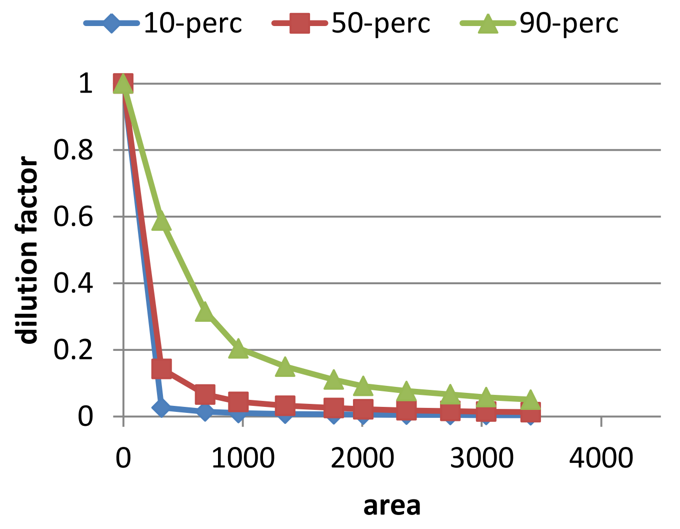

“Off-field Habitats” medium quality

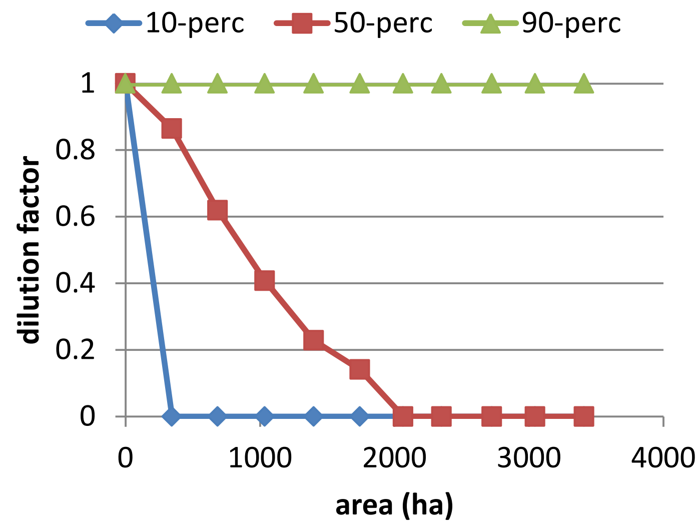

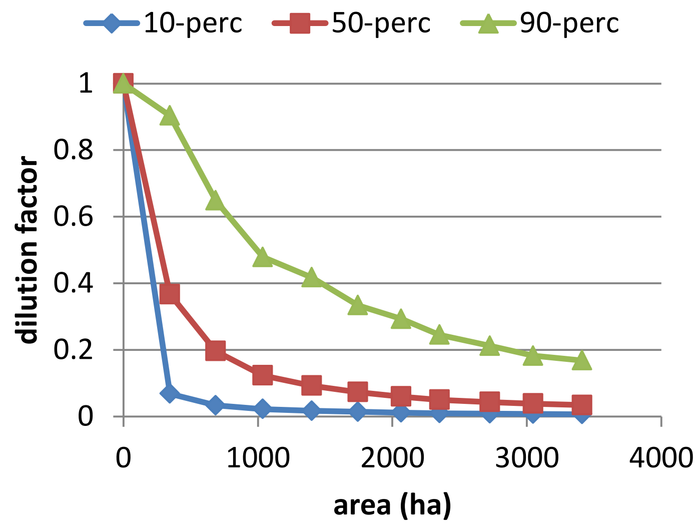

“Off-field Habitats” low quality

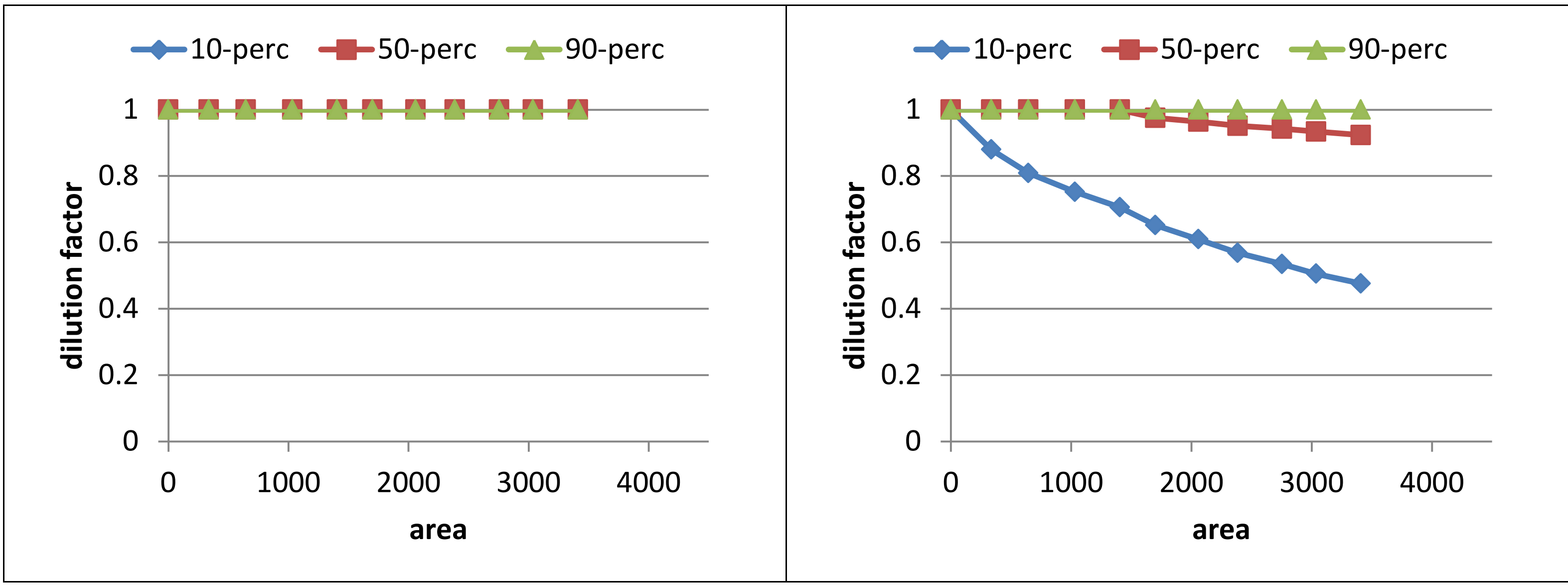

Figure S9. The 10-, 50- and 90-percentile dilution factor depending on the surface area of flower strips or off-field habitats. Rows from top to bottom: “flower strips” (1, 2, 5 and 10 m wide), “off-field habitats” (high, medium, low quality).

<table>
<tr><td>Competition SO-model</td><td>Competition RL-model</td></tr>
<tr><td colspan="2">“Alternative Fields”</td></tr>
<tr><td>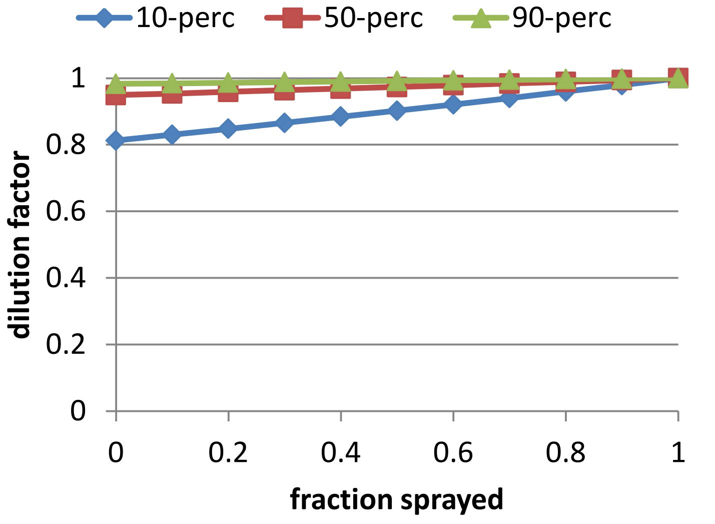
</td><td>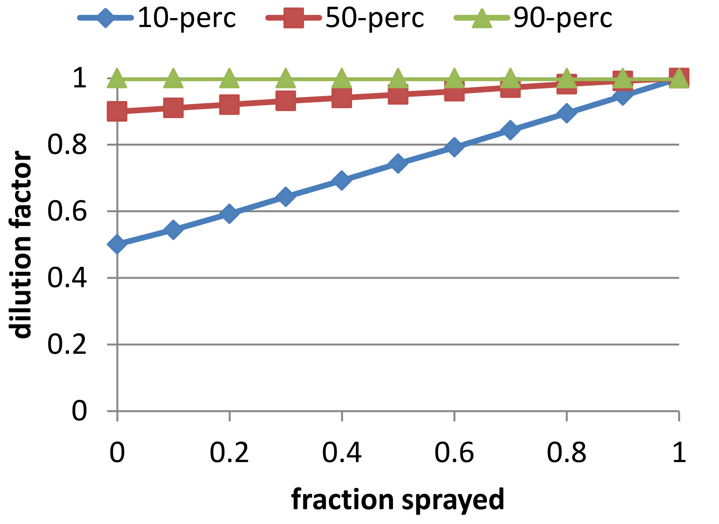
</td></tr>
<tr><td>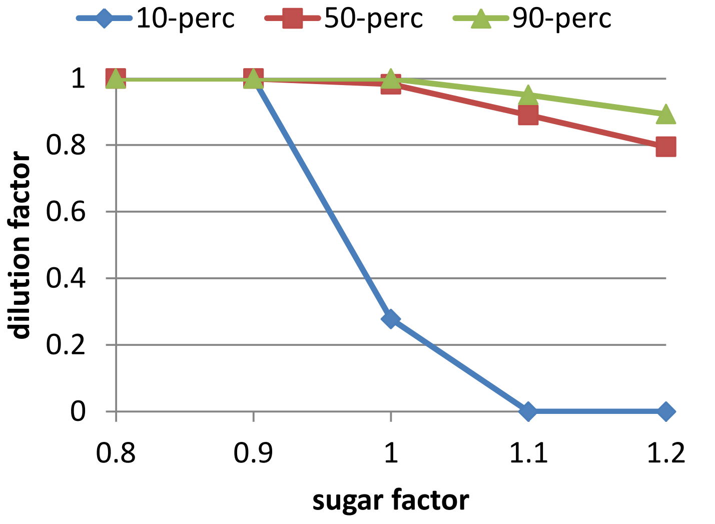
</td><td>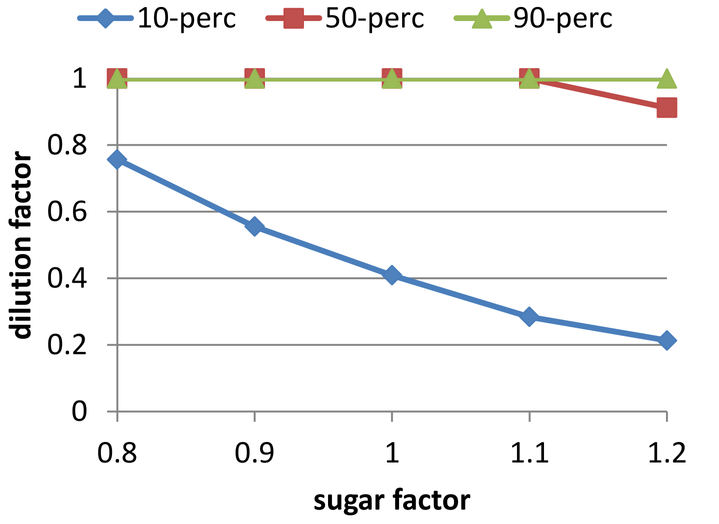
</td></tr>
<tr><td colspan="2">“Flower Strips” 1, 2, 5 and 10 m wide</td></tr>
<tr><td>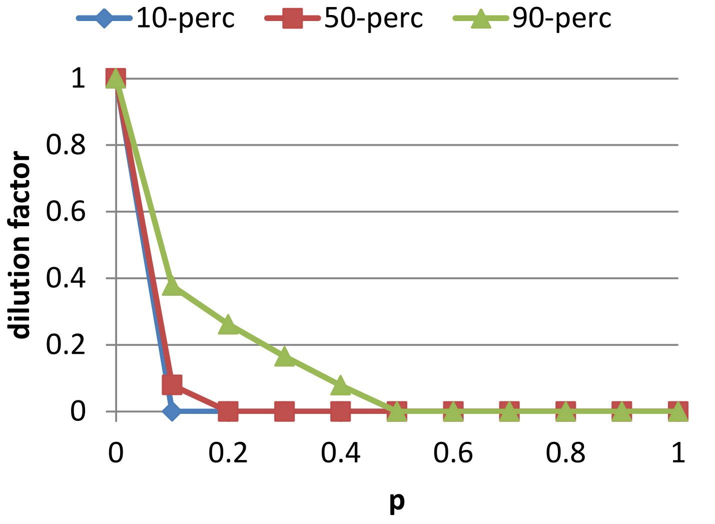
</td><td>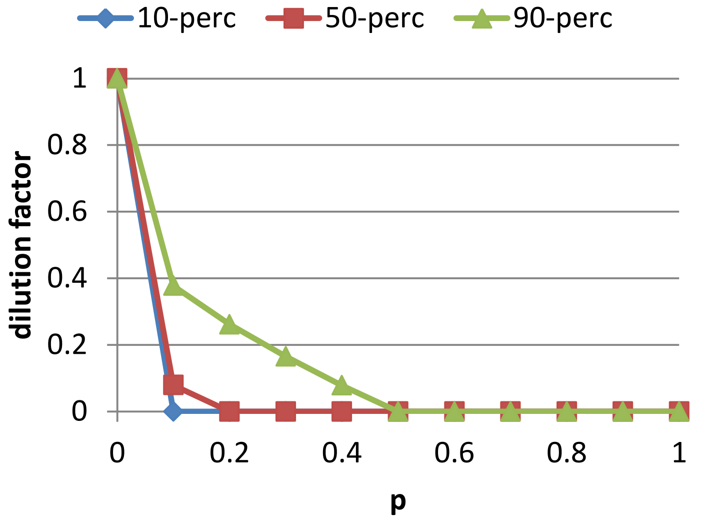
</td></tr>
</table>

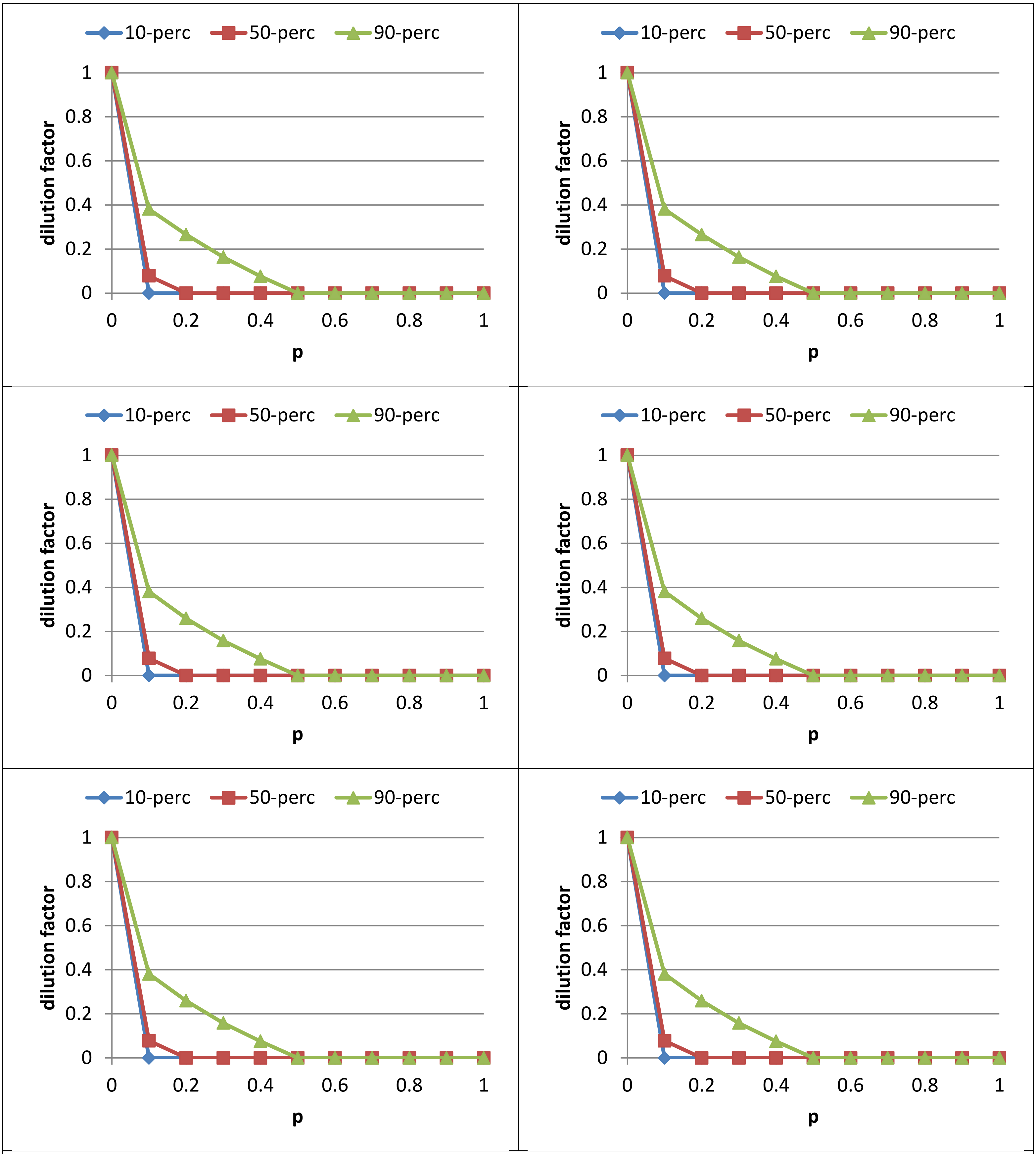

“Off-field Habitats” high, medium and low quality

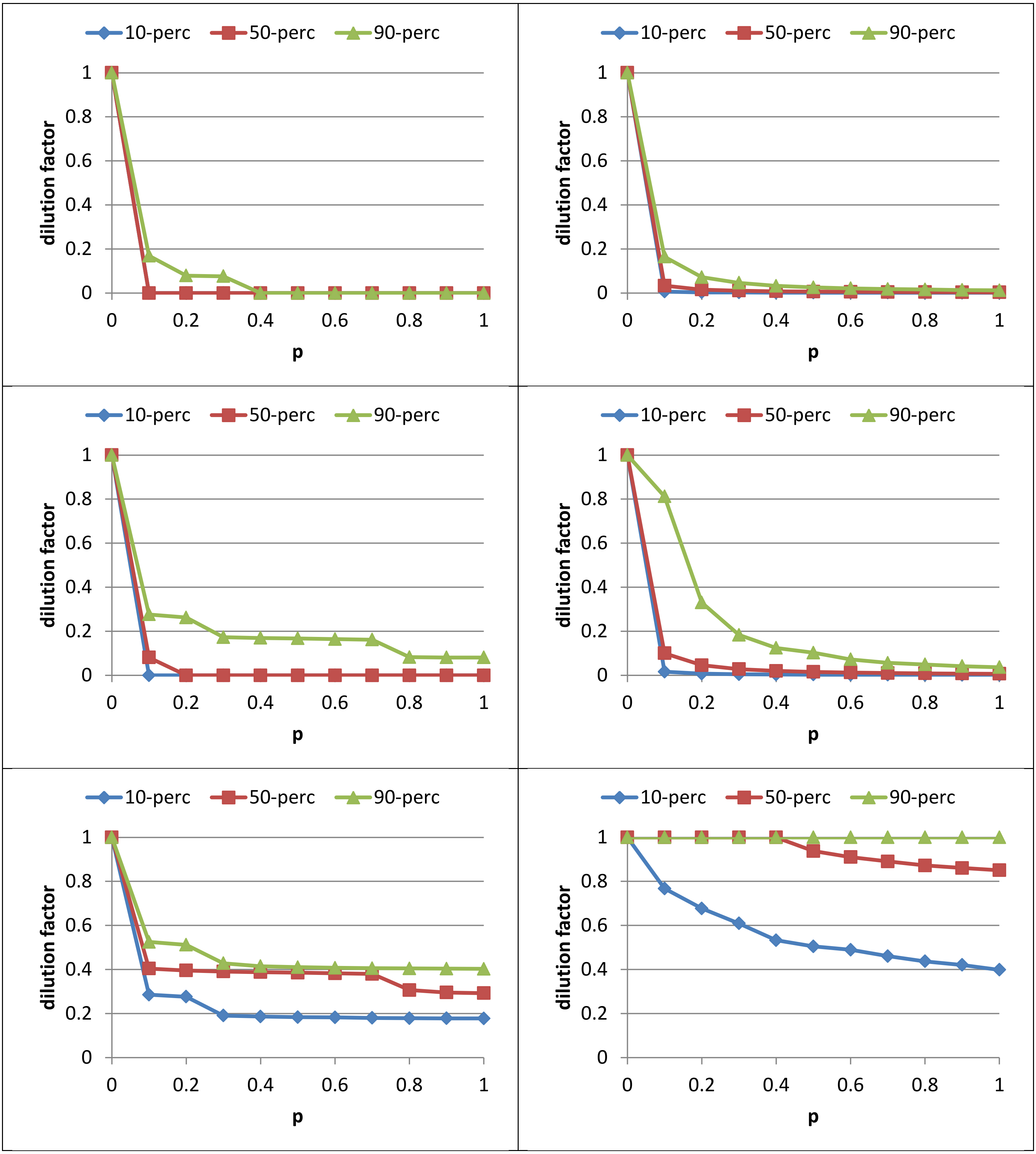
10-perc
50-perc
90-perc
dilution factor
p
0
0.2
0.4
0.6
0.8
1

Figure S10. The 10-, 50- and 90-percentile dilution factors for all scenarios, with constant competitor density of 0.1 m^{-2}, for the SO-model (left) and the RL-model (right). Rows from top to bottom: “alternative fields” (2x), “flower strips” (1, 2, 5, 10 m width), “off-field habitats” (high, medium, low quality).

SO-model competition	RL-model competition
“Alternative Fields”	

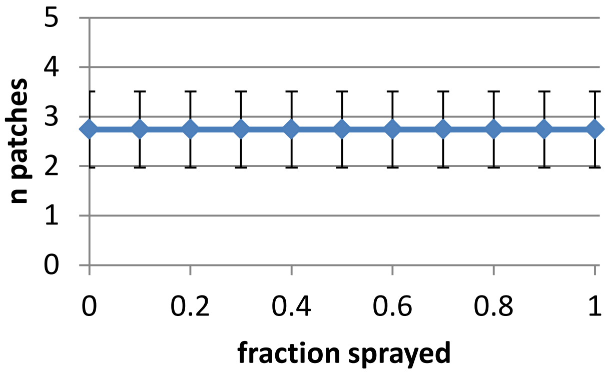

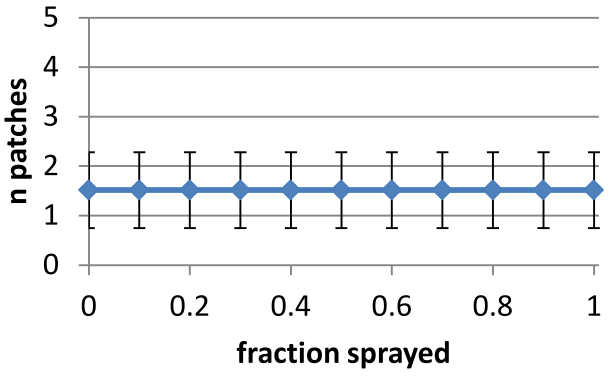

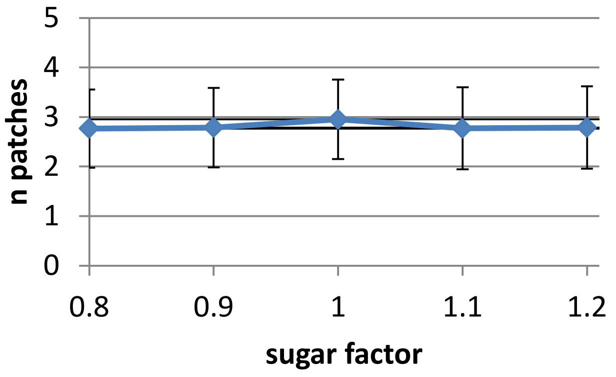

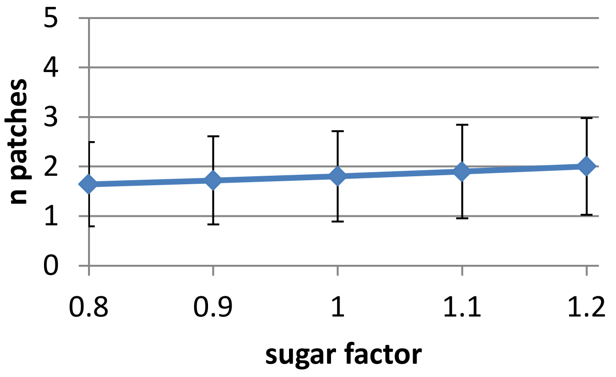

“Flower Strips” 1, 2, 5 and 10 m wide

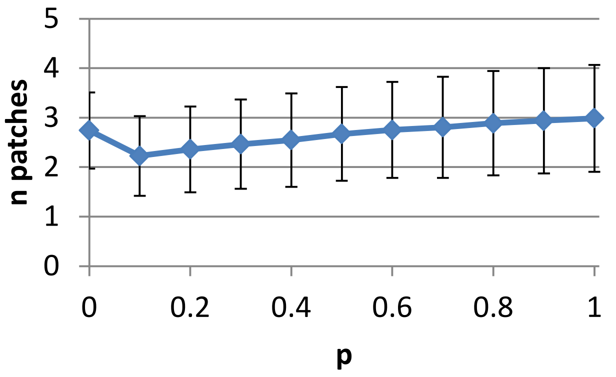

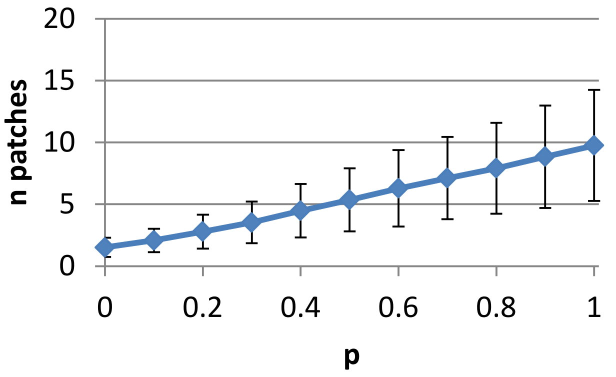

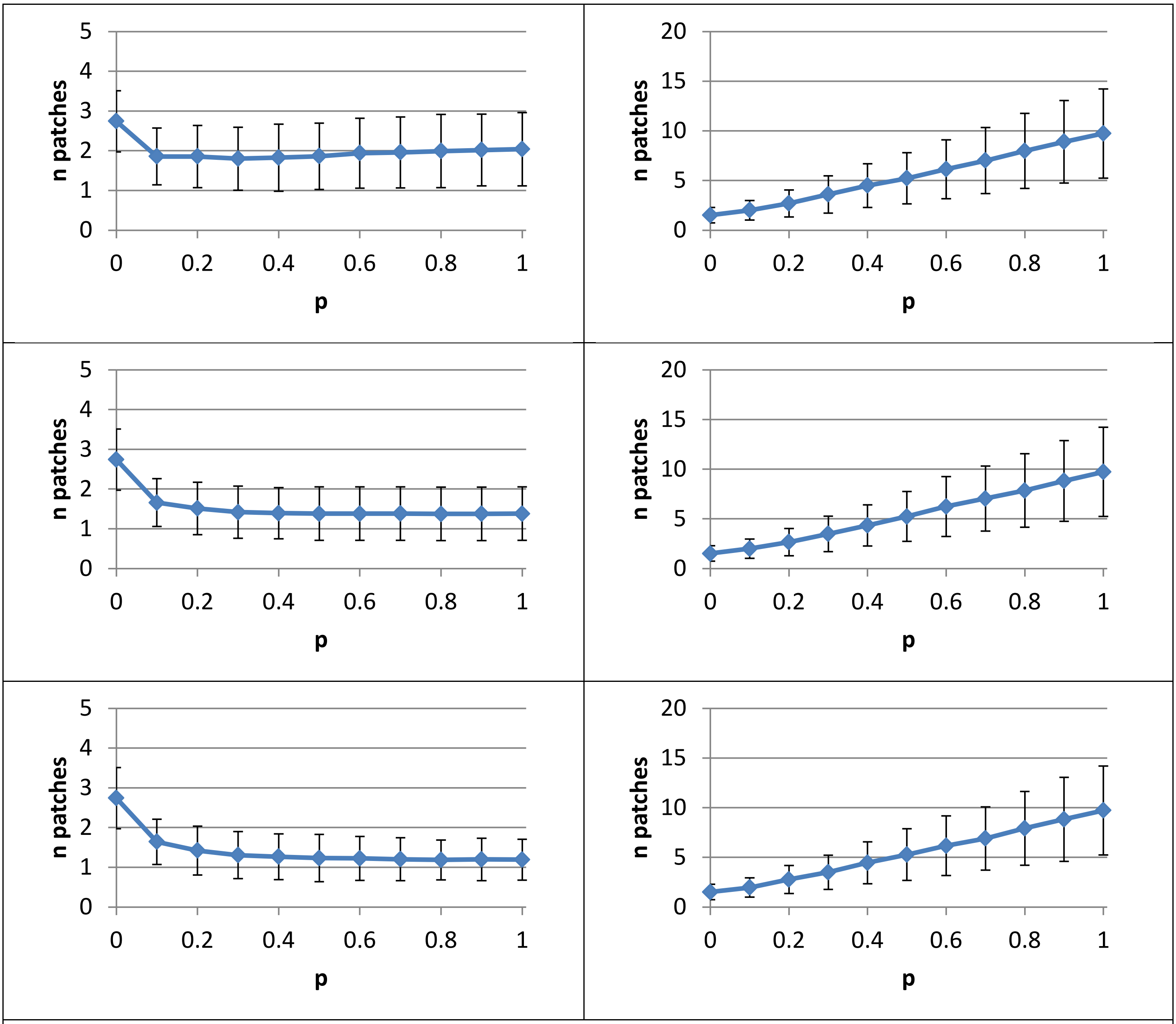

"Off-field Habitats" high, medium and low quality

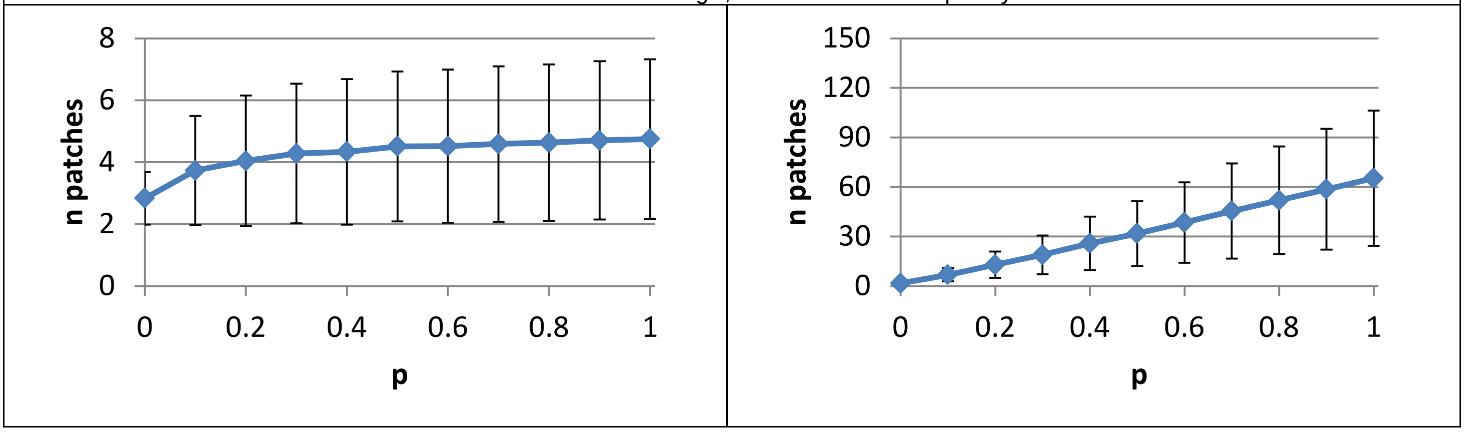

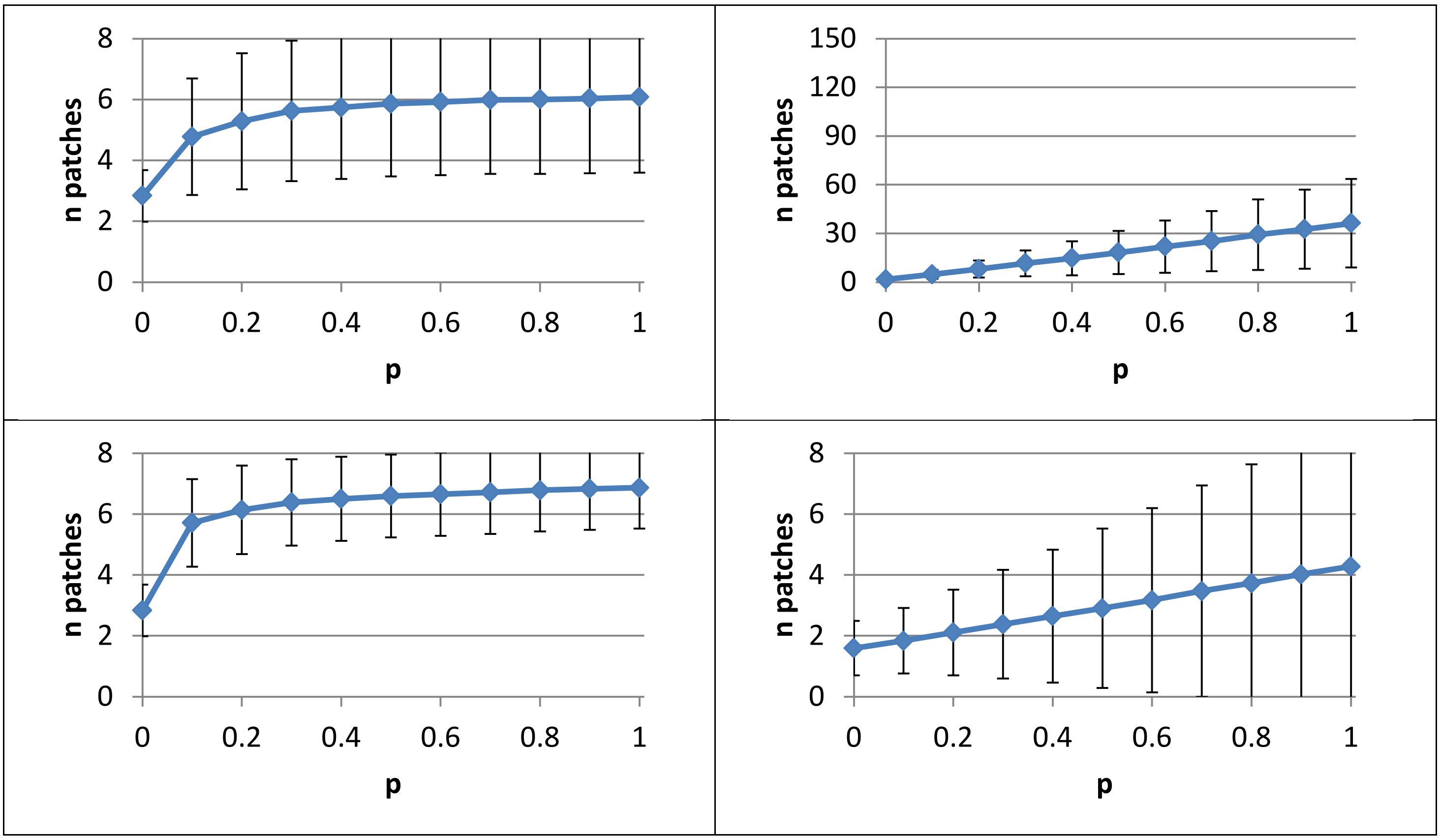

Figure S11. Number of patches exploited over the day in all scenarios with constant competitor density of 0.1 m^{-2}, for the SO-model (left) and the RL-model (right). Rows from top to bottom: “alternative fields” (2x), “flower strips” (1, 2, 5, 10 m width), “off-field habitats” (high, medium, low quality).

RL-model nr followers = 50	RL-model nr followers = 200

“Alternative Fields”

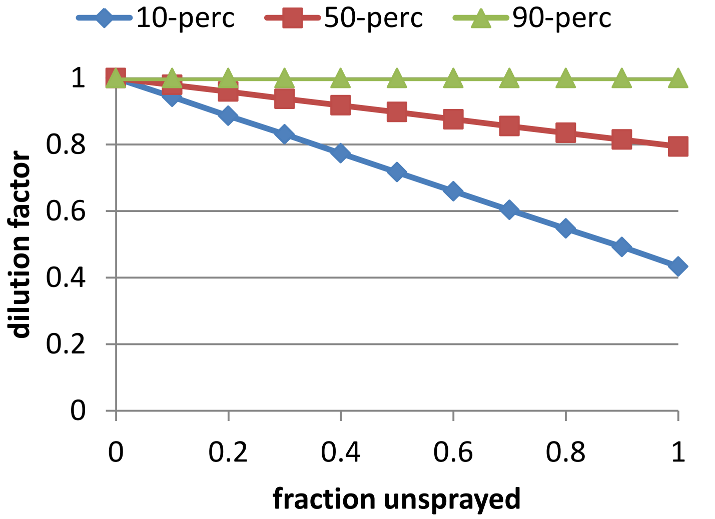

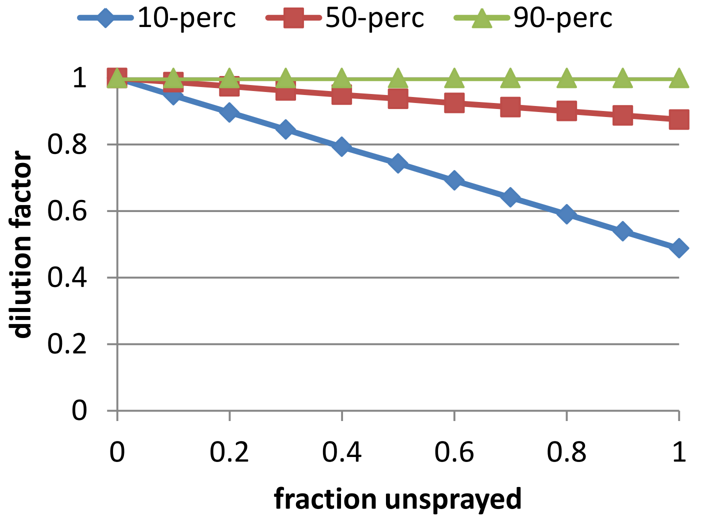

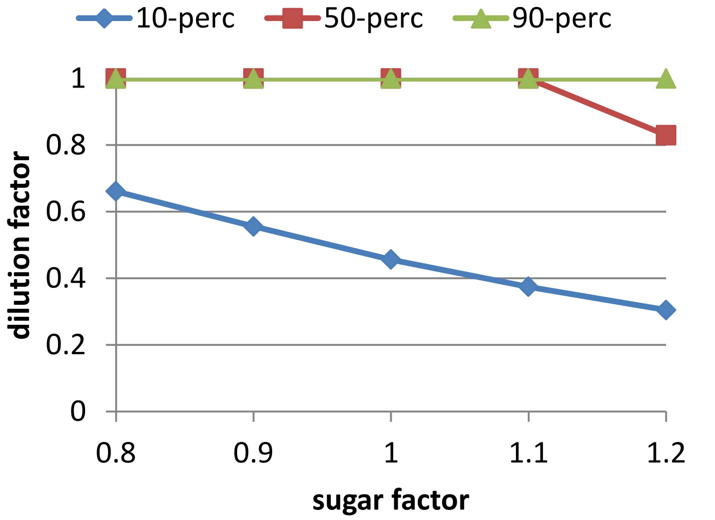

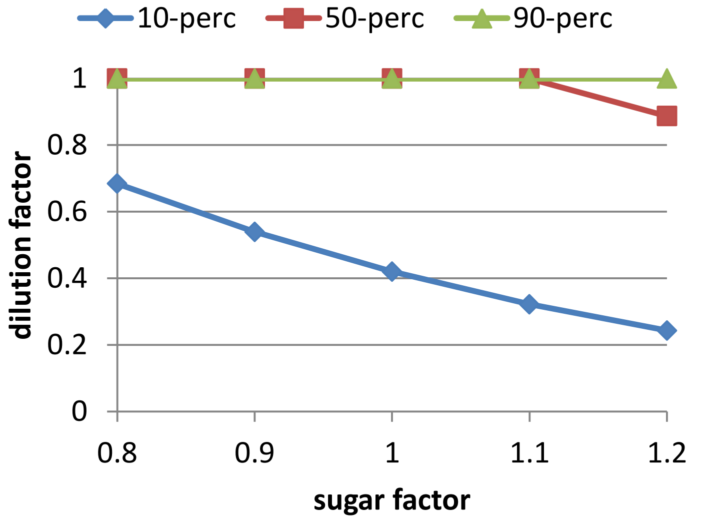

“Flower Strips” 1, 2, 5 and 10 m wide

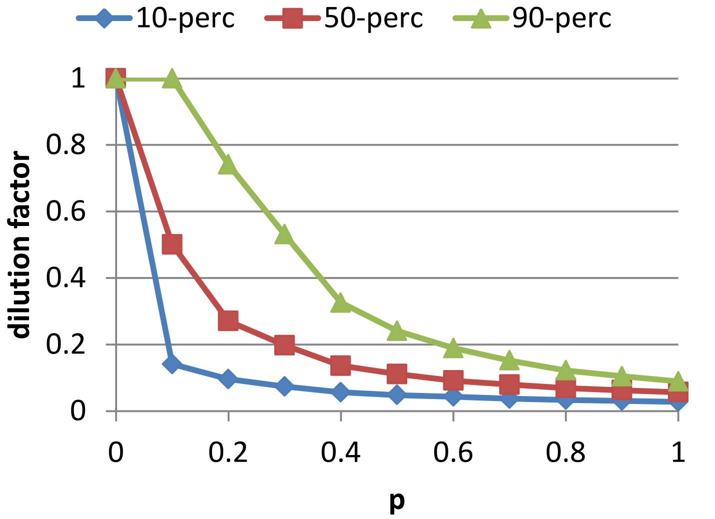

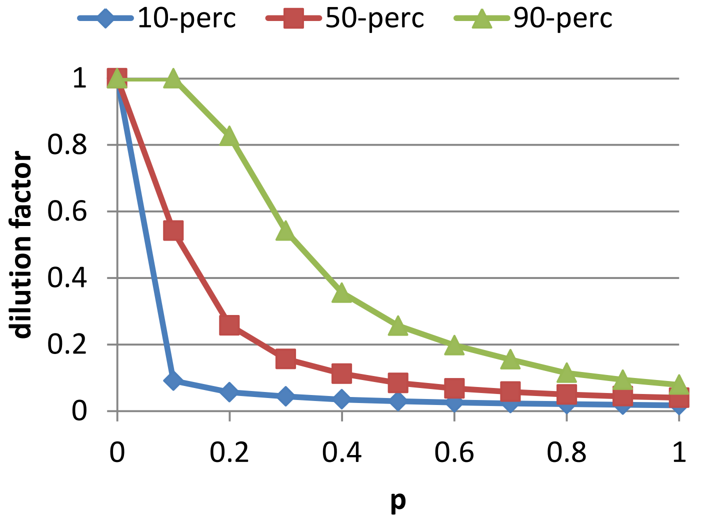

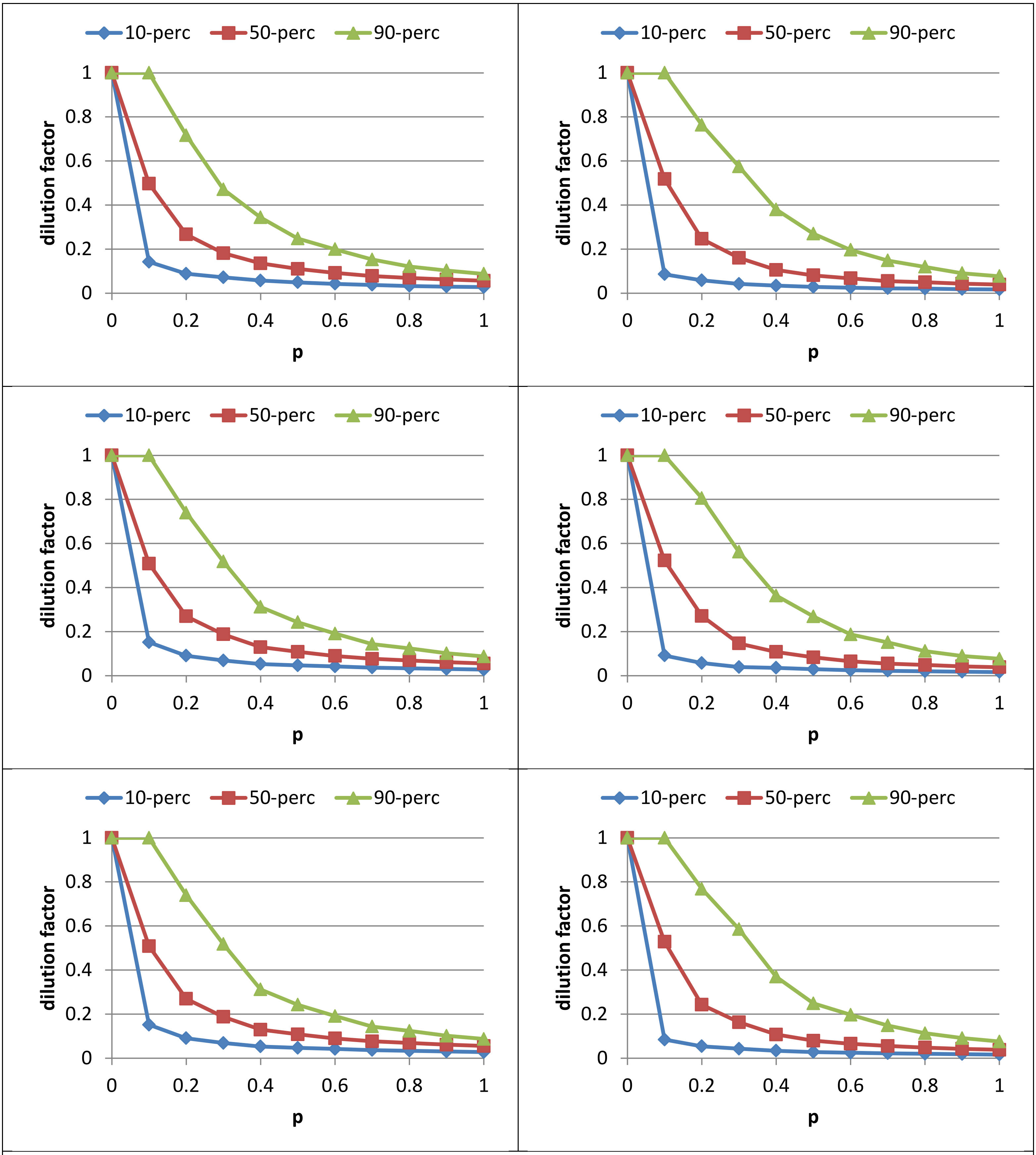

“Off-field Habitats” high, medium and low quality

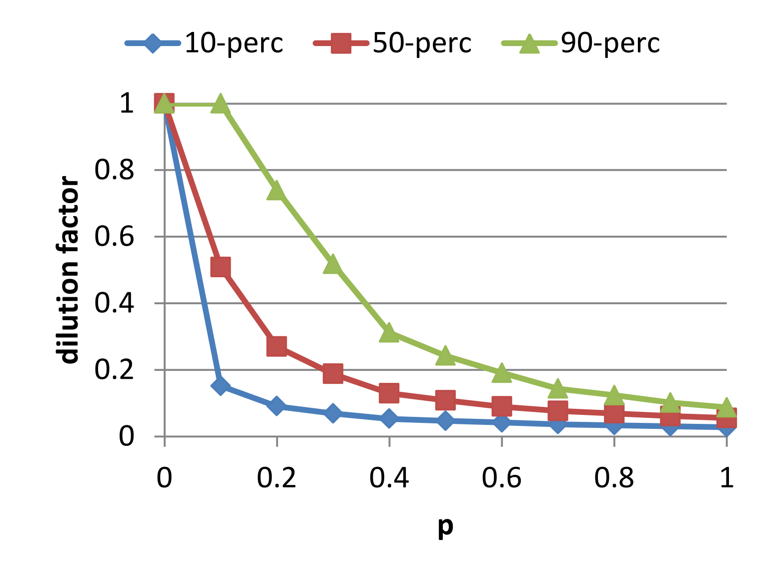
10-perc
50-perc
90-perc
dilution factor
1
0.8
0.6
0.4
0.2
0
0
0.2
0.4
0.6
0.8
1
p

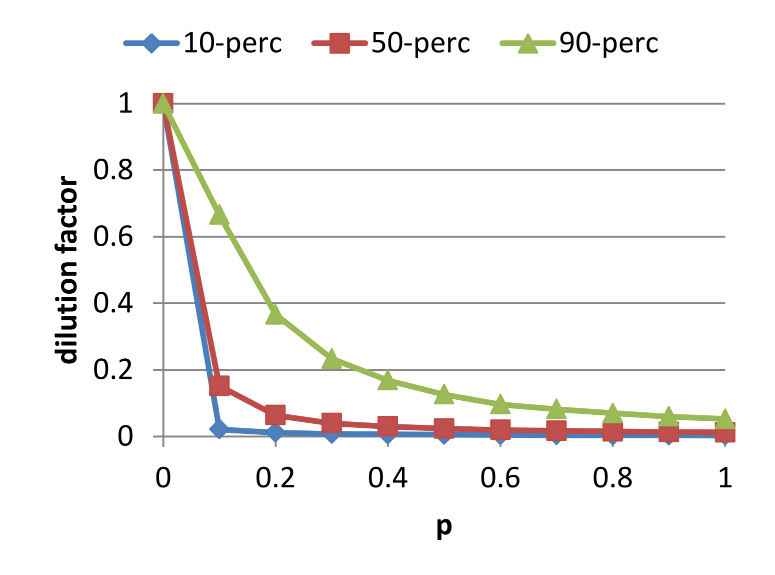
10-perc
50-perc
90-perc
dilution factor
1
0.8
0.6
0.4
0.2
0
0
0.2
0.4
0.6
0.8
1
p

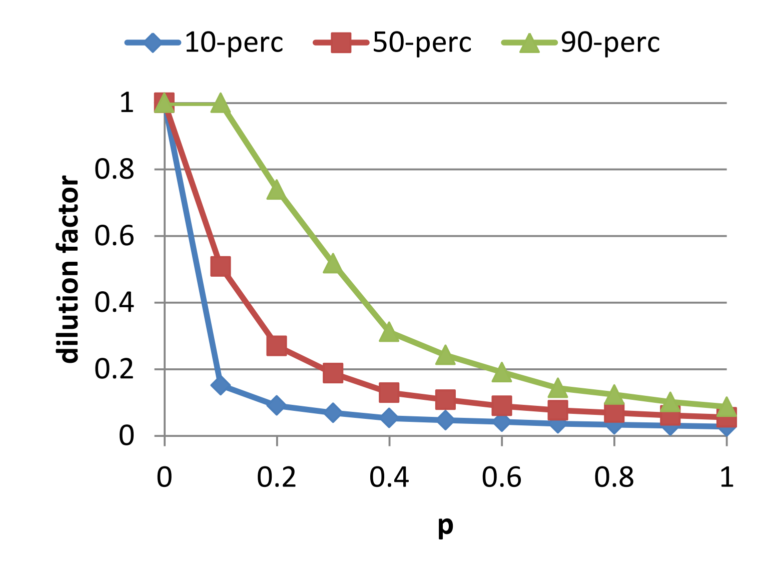
10-perc
50-perc
90-perc
dilution factor
1
0.8
0.6
0.4
0.2
0
0
0.2
0.4
0.6
0.8
1
p

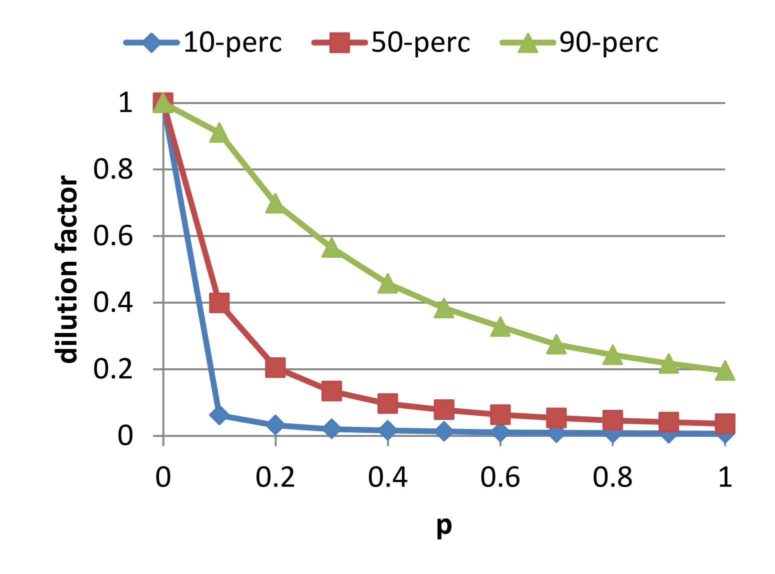
10-perc
50-perc
90-perc
dilution factor
1
0.8
0.6
0.4
0.2
0
0
0.2
0.4
0.6
0.8
1
p

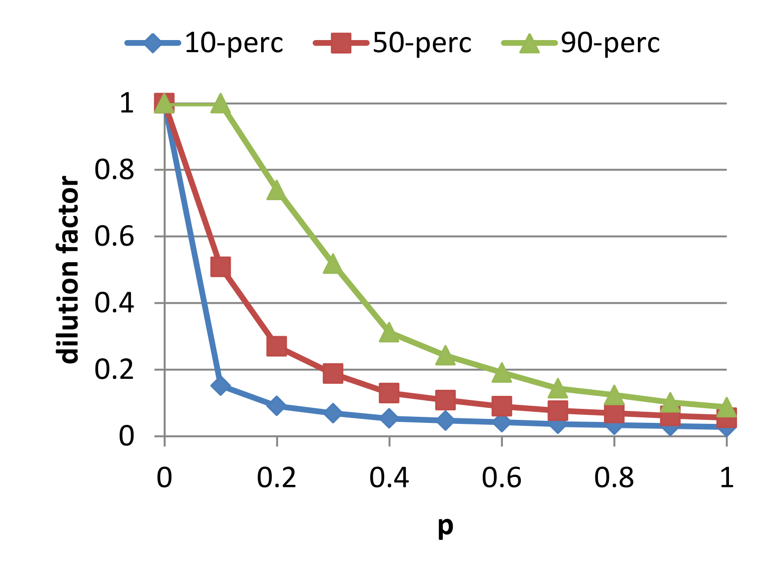
10-perc
50-perc
90-perc
dilution factor
1
0.8
0.6
0.4
0.2
0
0
0.2
0.4
0.6
0.8
1
p

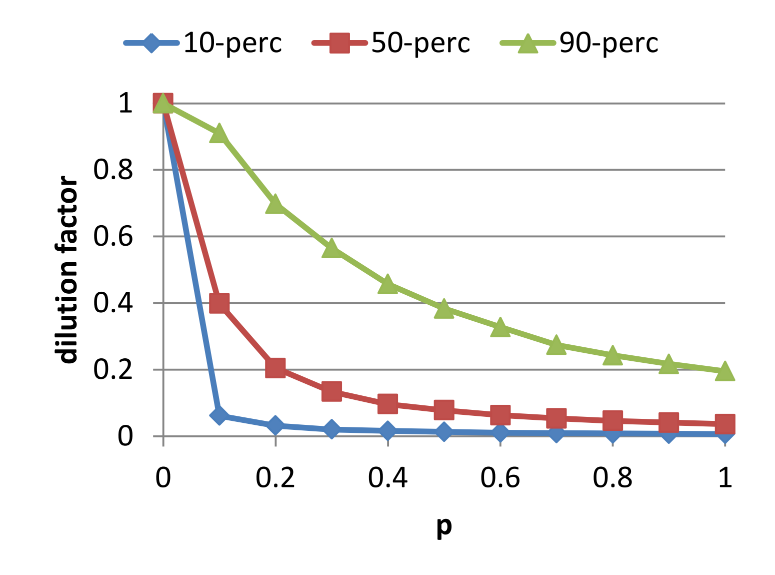
10-perc
50-perc
90-perc
dilution factor
1
0.8
0.6
0.4
0.2
0
0
0.2
0.4
0.6
0.8
1
p

Figure S12. The 10-, 50- and 90-percentile dilution factor in all scenarios for RL-model with the hourly number of followers set to 50 (left) or 200 (right) instead of 100 (default). Rows from top to bottom: “alternative fields” (2x), “flower strips” (1, 2, 5, 10 m width), “off-field habitats” (high, medium, low quality).

Nr followers: 50	Nr followers: 200

“Alternative Fields”

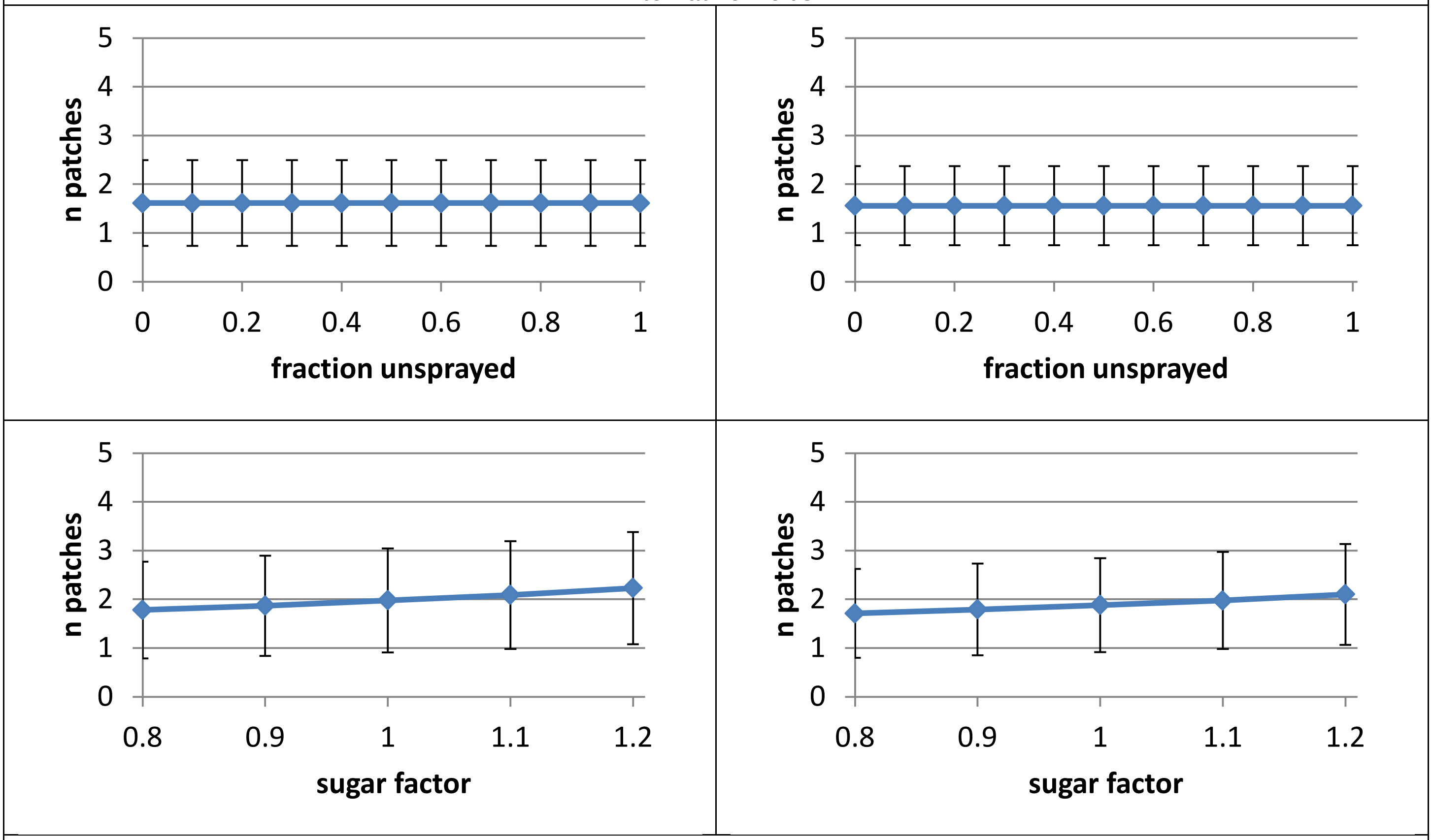

“Flower Strips” 1, 2, 5 and 10 m wide

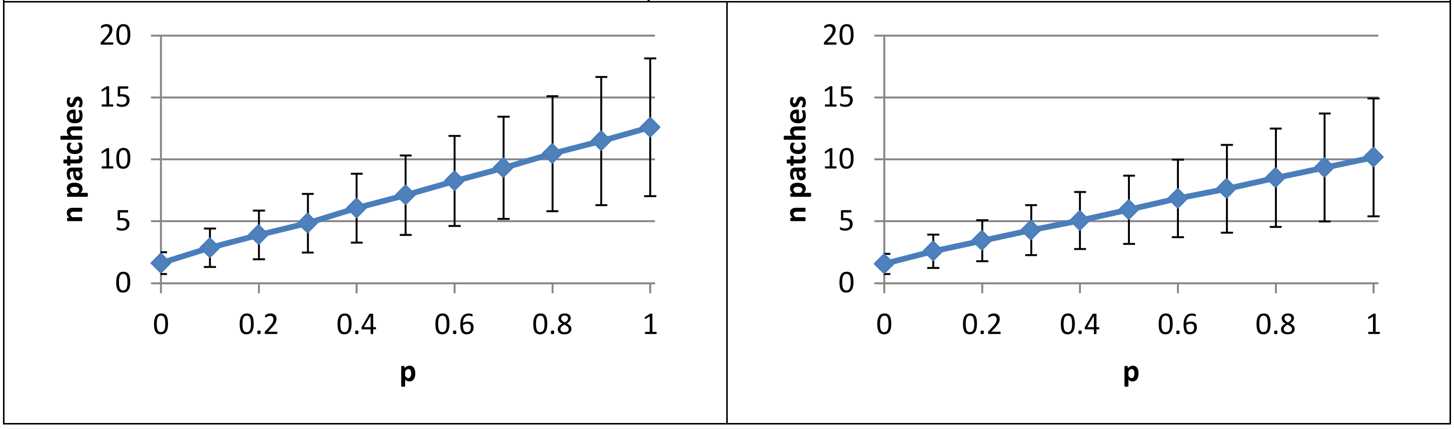

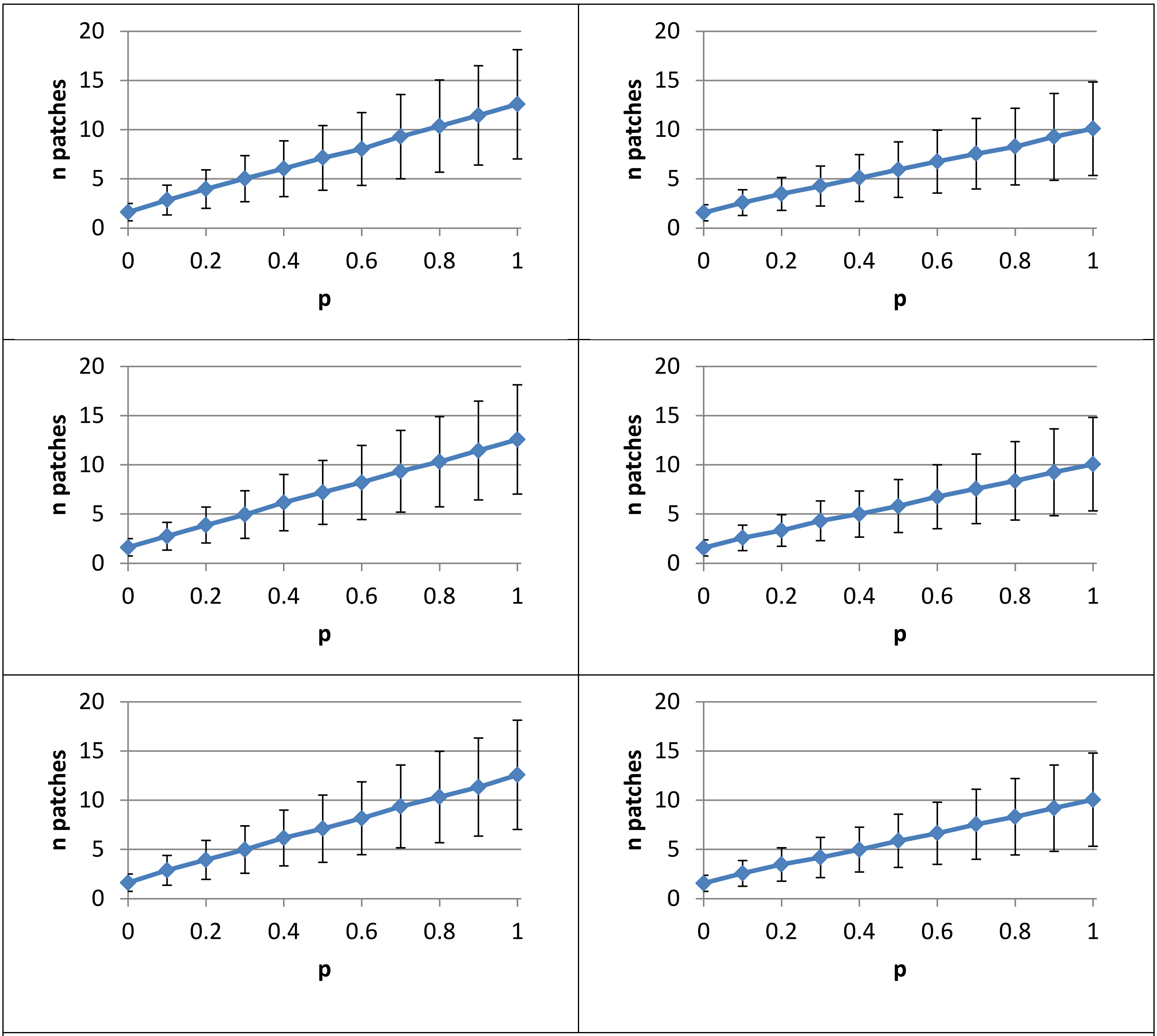

“Off-field Habitats” high, medium and low quality

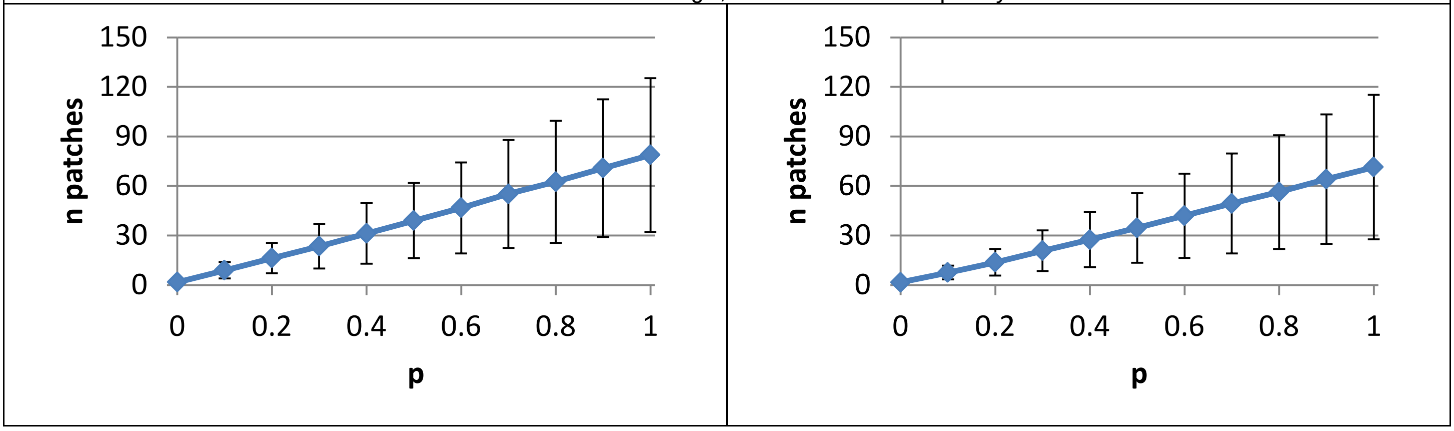

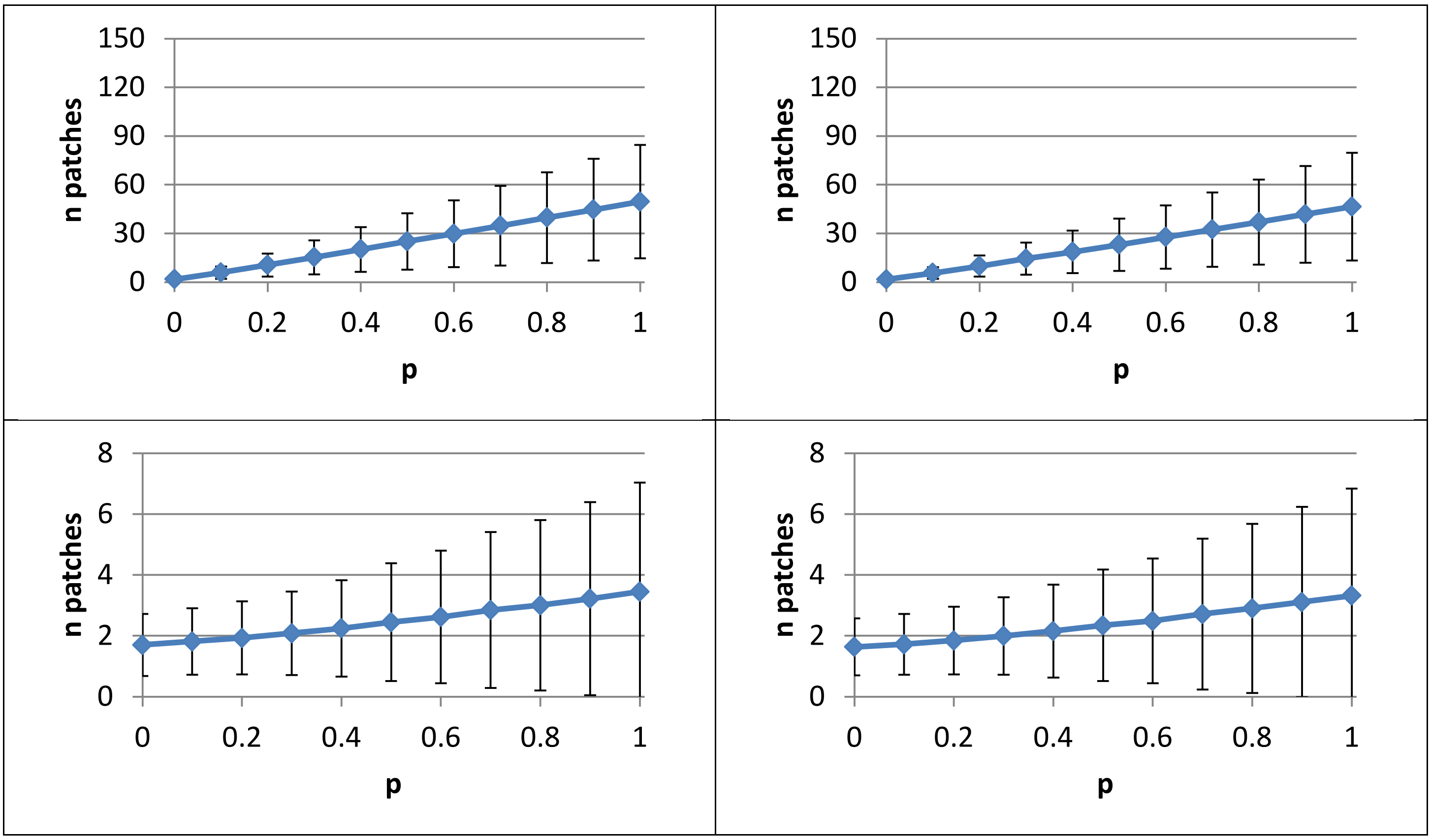

Figure S13. Number of patches exploited over the day in all scenarios for RL-model with the hourly number of followers set to 50 (left) or 200 (right) instead of 100 (default). Rows from top to bottom: “alternative fields” (2x), “flower strips” (1, 2, 5, 10 m width), “off-field habitats” (high, medium, low quality).
